# Cell-free fat extract improves ovarian function and fertility in mice with premature ovarian insufficiency

**DOI:** 10.1186/s13287-022-03012-w

**Published:** 2022-07-16

**Authors:** Mengyu Liu, Dan Zhang, Xiaowei Zhou, Jingru Duan, Yanqin Hu, Wenjie Zhang, Qiang Liu, Bufang Xu, Aijun Zhang

**Affiliations:** 1 Reproductive Medical Center, Department of Obstetrics and Gynecology, Ruijin Hospital, Shanghai Jiao Tong University School of Medicine, 197 Ruijin 2nd Road, Shanghai, 200025 China; 2 Shanghai Key Laboratory for Assisted Reproduction and Reproductive Genetics, Shanghai, 200135 China; 3 Shanghai Key Laboratory of Reproductive Medicine, Department of Histoembryology, Genetics and Developmental Biology, Shanghai Jiao Tong University School of Medicine, 280 South Chongqing Road, Shanghai, 200025 China; 4 Department of Plastic and Reconstructive Surgery, Shanghai 9th People’s Hospital, Shanghai Jiao Tong University School of Medicine, Shanghai Key Laboratory of Tissue Engineering, Shanghai, 200011 China

**Keywords:** Premature ovarian insufficiency, Cell-free fat extract, Fertility, Biosecurity, Granulosa cells

## Abstract

**Background:**

Premature ovarian insufficiency (POI) is a refractory disease that seriously affects the reproductive health of women and is increasing in incidence and prevalence globally. There is enormous demand to improve fertility in women with POI, while there is still lack of effective therapeutic methods in clinic. Cell-free fat extract (CEFFE) has been reported to contain thousands of active proteins which possess the ability to promote tissue repair in other diseases. In our study, we aimed to observe the efficacy and biosecurity of CEFFE on the repair of ovarian function and fertility of mice with POI and further explore the underlying mechanism.

**Methods:**

In vivo, POI mice model, established by cyclophosphamide (CTX, 120 mg/kg) and busulfan (BUS, 12 mg/kg), was treated with CEFFE via the tail vein every two days for 2 weeks. Then, the weight of ovaries, estrous cycle and follicle count by H&E staining were measured. The content of AMH, E_2_ and FSH in serum was measured by Enzyme-linked immunosorbent assay. Fertility was evaluated by the number of oocytes retrieved, the development of embryos in vitro and the litter size. Biosecurity of parent mice and their pups were examined by body mass and visceral index. The proliferation and apoptosis of cells in ovaries were examined by immunohistochemistry and transmission electron microscopy. Furthermore, the mRNA-Seq of mouse ovarian granulosa cells was performed to explore underlying mechanism of CEFFE. In vitro, KGN cell line and human primary ovarian granulosa cells (hGCs) were treated with 250 μM CTX for 48 h with/without CEFFE. The proliferative ability of cells was detected by cell counting kit-8 assay (CCK-8) and EDU test; the apoptosis of cells was detected by TUNEL and flow cytometry.

**Results:**

CEFFE recovered the content of AMH, E_2_ and FSH in serum, increased the number of follicles and the retrieved oocytes of POI mice (*P* < 0.05). CEFFE contributed to the development of embryos and improved the litter size of POI mice (*P* < 0.05). There was no side effect of CEFFE on parent mice and their pups. CEFFE contributed to the proliferation and inhibited the apoptosis of mouse granulosa cells in ovary, as well as in human ovarian granulosa cells (including KGN cell line and hGCs) (*P* < 0.05).

**Conclusions:**

The treatment of CEFFE inhibited the apoptosis of granulosa cells and contributed to the recovery of ovarian function, as well as the fertility of mice with POI.

**Supplementary Information:**

The online version contains supplementary material available at 10.1186/s13287-022-03012-w.

## Introduction

Premature ovarian insufficiency (POI) refers to a decline in ovarian function before the age of 40, which is often accompanied by inadequate estrogen (E_2_), elevated follicle stimulating hormone (FSH) and menstrual disorders and is characterized by exacerbated follicular atresia and progressive degeneration in oocyte quality, eventually leading to a sharp decline in fertility [1]. In addition to genetic, environmental and iatrogenic factors, 70–90% of idiopathic POI cases have no specific etiology [2, 3]. Hypofunction of ovarian reserves and poor oocyte quality were the most important cause for the low pregnancy of those patients with POI [4, 5]. The latest meta-analysis showed that the incidence of POI in women was as high as 3.7% [6]; moreover, cumulative incidence has also increased gradually [7, 8]. Unlike women with advanced age, they have a strong desire to get pregnant. Hence, it is urgent to find a new and effective strategy to solve this reproductive challenge for women with POI.

Recently, with the development of regenerative medicine, stem cell therapy has been studied for the treatment of POI [9]. Adipose-derived mesenchymal stem cells (ADSCs) can secrete hundreds of cytokines, exosomes and other active substances that have been suggested to exert diverse biological effects [9–11]. It has been reported that ADSCs can repair the ovarian function of POI mice through paracrine pathways, such as the release of VEGF, BDNF and other growth factors [12]. Further research showed that the conditioned medium of ADSCs could promote tissue regeneration in other diseases [13]. However, the use of stem cells in the clinic is limited and has some drawbacks, such as the insufficiency of autologous tissue, low cell retention/engraftment rates, tumorigenicity and immunogenicity risk, and inadequate off-the-shelf feasibility [14].

Cell-free fat extract (CEFFE) is a cell-free liquid component in which fat tissue is emulsified and centrifuged to remove oil, cells and extracellular matrix, resulting in an extract containing TGFβ, VEGF, EGF and other growth factors which were similar to those produced by some stem cells [15]. CEFFE has the advantages of low immunogenicity and rich sources. Previous studies have demonstrated that CEFFE can promote cell proliferation and angiogenesis and inhibit apoptosis in skin regeneration [16, 17], suggesting a potential effect on tissue repair and regeneration, but its effects on ovarian injury are largely unknown. To investigate the therapeutic and transformation potential of CEFFE on POI, a POI mouse model was constructed to explore the efficacy, safety and underlying molecular mechanism of CEFFE in restoring ovarian function and fertility, which would provide a novel approach for the treatment of POI in the clinic.

## Materials and methods

### Preparation of CEFFE

CEFFE was provided by SEME Cell Technology Co., Ltd. (Shanghai, China) and extracted as previously described [15]. Briefly, the sample was washed with saline several times, and then the clean fat layer was collected, mechanically emulsified and centrifuged to obtain a liquid layer. Finally, the liquid underwent a freeze–thaw process, followed by centrifugation to obtain the liquid layer, which was stored at − 80 °C. The protein concentration of CEFFE was measured by a BCA protein kit (Vazyme, China) and adjusted to 3 μg/μL.

### Establishment and treatment of POI model mice

**Fig. 1:**
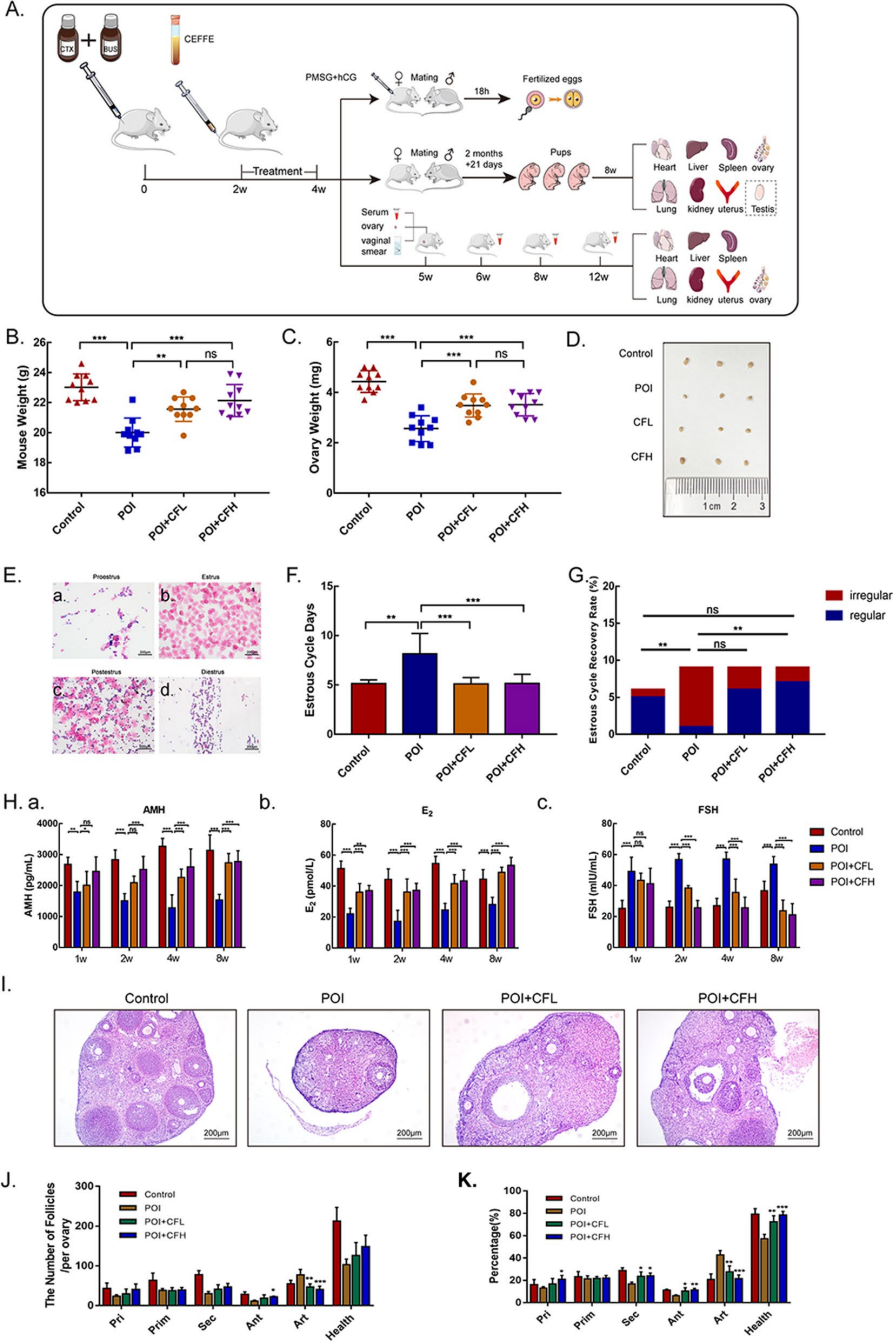
CEFFE improved ovarian function in POI mice. **A** Schematic design of the animal experiments. **B** The body weights of mice were measured 2 weeks after treatment with CEFFE (*n* = 10). **C** The ovary weights of mice were measured 2 weeks after treatment with CEFFE (*n* = 10). **D** Representative images of ovaries from the control, POI, POI + CFL and POI + CFH groups. **E** Typical morphology of vaginal smears showing different stages of the estrous cycle. a. Proestrus b. Estrus. c. Postestrus. d. Diestrus. Scale bars = 200 μm. **F** The average length of the estrous cycle (*n* = 9). **G** The ratios of regular and irregular estrous cycles of mice in each group (*n* = 9). **H** The levels of AMH, E_2_ and FSH were measured by ELISA at 1, 2, 4 and 8 weeks after treatment with CEFFE (*n* = 5). **I** The pathomorphology of ovaries was analyzed by H&E staining**.** Scale bars = 200 μm. **J** Quantitative analysis of follicles at different levels in total ovary (*n* = 4). **K** The percentage of different follicles in per ovary was analyzed (*n* = 4). Pri: Primordial follicles, Prim: Primary follicles, Sec: Secondary follicles, Ant: Antral follicles, Health: total number of health follicles (including primordial, primary, secondary and antral follicles), Atr: atretic follicles. All data are represented as the mean ± SD. ns: *P* > 0.05 represent the compare between two group which was labelled with short line above; *P < 0.05, **P < 0.01, ***P < 0.001, compared with POI group

Six- to eight-week-old female and male C57BL/6N mice were purchased from and feed in Shanghai Branch of Beijing Vital River Laboratory Animal Technologies (Shanghai, China). All experiments and procedures were approved by the Animal Research Committee of Vital River Laboratory Animal Technologies (IACUC-P2022002 authorization) according to the Guide for the Care and Use of Laboratory Animals (US National Institutes of Health publication, 8th edition, 2011). POI mice model was established with cyclophosphamide (CTX, Sigma–Aldrich, 120 mg/kg) and busulfan (BUS, Sigma–Aldrich, 12 mg/kg) by single intraperitoneal injection according to above methods (Additional file 1: Methods; Additional file 2: Fig. S1). The control group was injected with an equal volume of PBS. Then, the mice were injected with 200 μL of vehicle control (PBS, control and POI groups), 200 μL of CEFFE (1.5 μg/μL, POI + CFL group) or 200 μL of CEFFE (3 μg/μL, POI + CFH group) via the tail vein every two days for 2 weeks. The volume and total protein of CEFFE were referred to the principle of intravenous injection in mice and our previous research [18]. Finally, the mice were mated with male mice for fertility analysis or sacrificed for further experiments. The detailed process is illustrated in the schematic diagram of the animal experiment (Fig. 1A).

### Vaginal smears of mice

Vaginal smears were performed at 9:00 am every morning for 14 days to examine the estrous cycle after treatment with CEFFE. First, cotton swabs were moistened with saline, rotated gently in the vagina, and then smeared on adhesive slides. Next, the detached cells were stained with hematoxylin and eosin (H&E) to determine the different stages of the estrous cycle, such as proestrus, estrus, postestrus and diestrus.

### Enzyme-linked immunosorbent assay (ELISA) of hormones

The blood was collected when the mice were sacrificed and stored at room temperature for 30 min. Then, the samples were centrifuged at 2500 rpm for 15 min to obtain the serum, and the levels of anti-Mullerian hormone (AMH), E_2_, and FSH were measured with an ELISA kit (Mlbio, China) according to the manufacturer’s instructions. Briefly, 30 μL of serum was added to each coated well, incubated for 60 min, and then washed five times. After the samples were incubated with HRP-conjugated antibodies and the Solution A and B mixture, 50 μL of stop solution was added to stop the reaction. Finally, the optical density (OD) was measured at a wavelength of 450 nm, and the concentration was calculated based on the standard curve.

### Assessment of ovarian follicle counts

Ovaries were collected from each mouse after 2 weeks of treatment with CEFFE. After being fixed with 4% paraformaldehyde, the ovaries were embedded in paraffin and then serially sliced into 5 μm sections and stained with H&E. Follicles were counting every tenth section according to the different morphological characteristics to determine the follicle stage, such as primordial, primary, secondary, antral, and atretic follicles [19]. Then, the percentage of different follicles in per ovary was further analyzed.

### Embryo acquisition and culture

After 2 weeks of treatment with CEFFE, mice in the different groups were superovulated with 10 IU of pregnant mare serum gonadotropin (PMSG), followed by an intraperitoneal injection of 10 IU of human chorionic gonadotropin (hCG) 48 h later. Then, the female mice were immediately mated with aged-matched males with proven fecundity. After 18 h, oocytes or zygotes were collected from the ampullae of the already mated female mice and those were cultured in vitro. Meanwhile, the total number of oocytes retrieved was recorded. Next, the oocytes or zygotes were cultured in liquid drops containing 40 μL KSOM (Millipore) under mineral oil (Sigma) at 37 °C in a 5% CO_2_ atmosphere. After about 3 days, blastocysts were observed and recorded [20].

### Fertility assay

For natural mating, after being treated with CEFFE, two female mice were mated with one 8-week-old male with proven fecundity for two months. And the female mice didn’t receive the injection of PMSG or hCG in this test. When the female mice were obviously pregnant, they were individually caged to give birth, and the number of offspring was recorded. Two months later, the male mice were separated from the female mice for 21 days to further confirm whether the other female mice were pregnant.

### Self and offspring safety assessments

After 8 weeks of treatment with CEFFE, the ovarian tumorigenicity of CEFFE in parent mice was assessed by measuring the expression of the cancer markers PTEN (suppressive gene) and P53 (oncogenic gene) in the ovaries by immunohistochemical analysis. The mice were weighed and sacrificed by cervical dislocation. Next, organs were collected for weighing, including the heart, liver, spleen, lung, kidney, uterus and ovary, to analyze the organ index, which was defined as the ratio of organ mass and body mass. Similarly, the safety assessment of offspring mice was performed by monitoring the body weights of pups weekly and evaluating the organ index at 8 weeks after birth.

### Immunohistochemistry (IHC)

The ovarian sections were subjected to IHC. The steps were as follows: dewaxing and rehydrating the paraffin section, antigen retrieval, blocking endogenous peroxidase activity, serum sealing, primary antibody incubation, secondary antibody incubation, DAB chromogenic reaction, nuclear counterstaining and mounting. Detailed information on the primary antibodies are shown in Additional file 3: Table S1.

### Transmission electron microscopy (TEM)

Ovaries were fixed in TEM fixative (Servicebio, China) and then dehydrated in gradient alcohol and acetone at room temperature. After being polymerized with resin, the ovaries were cut to a thickness of 80 nm on an ultramicrotome. Finally, the samples were observed by TEM (HT7700, Hitachi, Japan).

### Cell culture of KGN cells and hGCs

The human ovarian granulosa-like tumor (KGN) cell line (Feiya Biotechnology Co., Ltd., China) and human primary ovarian cumulus granulosa cells (hGCs) were used for the in vitro study. Cumulus granulosa cells were collected from young women (aged < 40 years) with tubal factor or male factor infertility with informed consent, and the oocyte retrieval procedure was approved by the Ethics Committee of Ruijin Hospital (2020104a). The patients with abnormal chromosome, endometriosis, polycystic ovarian syndrome and other diseases which could affect folliculogenesis were excluded. hGCs were isolated from cumulus-oocyte complexes by mechanical stripping and then digested with hyaluronidase (1 mg/mL, Yeasen, China). Both KGN cells and hGCs were cultured in DMEM/F12 media (Thermo, USA) with 10% fetal bovine serum (Gibco, USA) and 1% penicillin and streptomycin in a humidified incubator at 37 °C with 5% CO_2_.

### Proliferation assay

KGN cell proliferation was assessed with a Cell Counting Kit (CCK-8, Yeasen, China). Cells were plated in a 96-well plate at a density of 2000 cells/well. On the second day, 10 µL of CCK-8 solution was added to each well. At different time points, the OD was measured using a microplate reader (Bio-Tek, China) at 450 nm.

The BeyoClickTM EdU-488 assay was performed according to the manufacturer’s protocol (Beyotime, China). Briefly, KGN cells or hGCs were cultured in 48-well plates for 48 h with 250 μM CTX and different concentrations of CEFFE. Then, proliferating cells were labeled with EdU, and the nuclei were labeled with Hoechst 33342. Finally, images of five random fields were captured by an inverted fluorescence microscope (Axio Vert.A1, Zeiss, Germany).

### Cell cycle assay

Cells were collected and fixed in 70% ethanol for 24 h at 4 °C. Then, the cells were incubated with propidium iodide and ribonuclease for 30 min at 37 °C in the dark. Flow cytometry was used to examine the cell cycle according to the standard process. Cell cycle distribution was analyzed by ModFit with cell cycle fitting software, and the proportions of G0/G1, S and G2/M were calculated.

### Immunofluorescence staining

Cells were fixed with 4% paraformaldehyde for 15 min, permeabilized and blocked. Next, the cells were incubated with primary antibodies at 4 °C overnight. The next day, the cells were incubated with a fluorescently labeled secondary antibody, and the nuclei were subsequently labeled with Hoechst 33342. Finally, images were taken by an inverted fluorescence microscope. Detailed antibody information is listed in Additional file 3: Table S1.

### Flow cytometry

Cell apoptosis was measured with an Annexin V APC/7-AAD apoptosis detection kit (Biolegend, USA). Cells were harvested by trypsinization without EDTA and suspended in 1 × binding buffer at a density of 5.0 × 10^6^cells/ml. Then, 5 μL of APC-conjugated Annexin V and 5 μL of 7-AAD were added to the cell suspension per 100 μL and incubated for 15 min in the dark at room temperature. Then, Annexin V Binding Buffer was used to stop the reaction. The samples were analyzed by flow cytometry (Beckman-Coulter, China).

### Mitochondrial membrane potential assay

To observe early apoptosis in cells, the mitochondrial membrane potential was examined with JC-1 (Beyotime, China) and flow cytometry or fluorescence microscopy according the manufacturer’s instructions. JC-1 monomers (green fluorescence) were detected with the FITC channel, and JC-1 aggregates (red fluorescence) were detected with the PE channel. Finally, the mean fluorescence intensity of red or green was analyzed, and the ratio of red to green was calculated.

### TUNEL assay

A TUNEL cell apoptosis detection kit (Beyotime, China) was used to examine apoptosis in ovarian sections and ovary granulosa cells (including KGN cells and hGCs). The analysis was performed according to the manufacturer’s instructions. In brief, after being permeabilized, the sample was incubated with the TUNEL detection solution. The nucleus was labeled with Hoechst 33342. Finally, the samples were analyzed by fluorescence microscopy (Axio Vert. A1, Zeiss, Germany).

### Collection of granulosa cells (GCs) from mouse ovaries

Ovaries were collected in DMEM/F12 medium 48 h after the mice were intraperitoneally injected with 10 IU of PMSG. Then, the follicles in the ovary were punctured with 30-gauge needles, and the oocytes were removed from the granulosa under a stereomicroscope. Finally, the GCs were collected, immediately stored in liquid nitrogen, and then stored at − 80 ℃ for further RNA-seq or RT–PCR analysis.

### RNA-Seq and bioinformatics analysis

RNA sequencing and bioinformatics analysis were performed at Shanghai Silver Crown Biomedical Technology Co., Ltd. First, total RNA was extracted from mouse GCs using a miRNeasy Mini Kit (Qiagen, USA), and then an Illumina NovaSeq 6000 (Illumina, USA) was used for RNA-Seq library preparation and sequencing. Each group consisted of 3 individual samples. Differentially expressed genes (DEGs) between each pair of groups were identified with the following criteria: |fold change|> 1.5 and *P* value < 0.05. The DEGs were further analyzed by Venn analysis and gene ontology (GO) and Kyoto encyclopedia of genes and genomes (KEGG) enrichment analysis.

### Statistical analysis

All experiments were performed at least three times. The data were analyzed by GraphPad Prism (software version 7.0) and are presented as the mean ± SD. Unpaired Student’s t test was used for comparisons between two groups. One-way ANOVA or two-way ANOVA was used for comparisons among multiple groups. A *P* value of < 0.05 was considered to be significantly different.

## Results

### Construction of the POI mouse model

To establish the POI model, the mice were intraperitoneally injected with different concentrations of CTX and BUS, and then various indicators were analyzed. Body weight was measured every two days, and ovary weight was measured after 14 days. Compared with those in the control group, the body and ovarian weights of mice in Groups 2, 3 and 4 decreased significantly, while there was no difference between Group 1 and the control group (Additional file 2: Fig. S1A–D). In addition, the sex hormone results demonstrated that the levels of AMH and E_2_ in Groups 2, 3 and 4 were significantly lower than those in the control group, but FSH was obviously increased (Additional file 2: Fig. S1E). Finally, H&E staining showed that the number of growth follicles in Groups 1, 2, 3 and 4 gradually decreased, the layer of granulosa cells was irregular, and the infiltration of inflammatory cells increased compared with those in the control group (Additional file 2: Fig. S1F, H). Moreover, ovarian granulosa cell apoptosis was measured by IHC analysis of Cleaved-Caspase3, and more apoptotic cells were observed in the four treatment groups than in the control group, especially in Groups 3 and 4 (Additional file 2: Fig. S1G, I). Based on these results, ovarian injury worsened gradually with increasing doses of CTX and BUS, but there was no significant difference between Group 3 and Group 4. Thus, Group 3 (120 mg/kg CTX + 12 mg/kg BUS) was selected to establish the POI model.

### Therapeutic effects of CEFFE on POI mice

To evaluate the effectiveness of CEFFE on POI mice, body weight, ovary weight, estrous cycle, sex hormones and ovarian morphology were analyzed at different time points. First, at the end of treatment, body weight in the POI group was obviously less than that in the control group, while mice in the POI + CFL and POI + CFH groups significantly gained weight (Fig. 1B). Similar results were observed for ovary weight (Fig. 1C, D; Additional file 2: Fig. S5). However, no difference in body or ovary weight was observed between the POI + CFL and POI + CFH groups. Second, vaginal smears were used to examine whether CEFFE could promote estrous cycle recovery according to different cell morphologies in vaginal smears [21] (Fig. 1E). Regular estrous cycles were observed in the control group and lasted for 4–6 days. Irregular and prolonged estrous cycles were observed in the POI group (Fig. 1F). The duration and the proportion of regular estrous cycles in the POI + CFH group were restored (Fig. 1F, G), but only the duration of the estrous cycle was restored in the POI + CFL group. In addition, the levels of E_2_ and AMH were significantly decreased in the POI group compared with control group, while those were significantly increased in the POI + CFL and POI + CFH groups compared with POI group. In contrast, the increased FSH in the POI group was reduced significantly in the POI + CFL and POI + CFH groups (Fig. 1H). These results suggested that CEFFE could rescue hormone secretion in the ovary, and the therapeutic effect was continuous and stable for at least 8 weeks. Finally, the pathological morphology and follicle counts of ovaries were observed by H&E staining. In the POI group, the percentage of primordial, primary, secondary and antral follicles were decreased. In the POI + CFL and POI + CFH groups, the percentage of healthy follicles (including primordial, primary, secondary and antral follicles) was increased, and atretic follicles were obviously decreased, especially the secondary and antral follicles (Fig. 1I–K).

### Rescue of POI mouse fertility after CEFFE injection

**Fig. 2:**
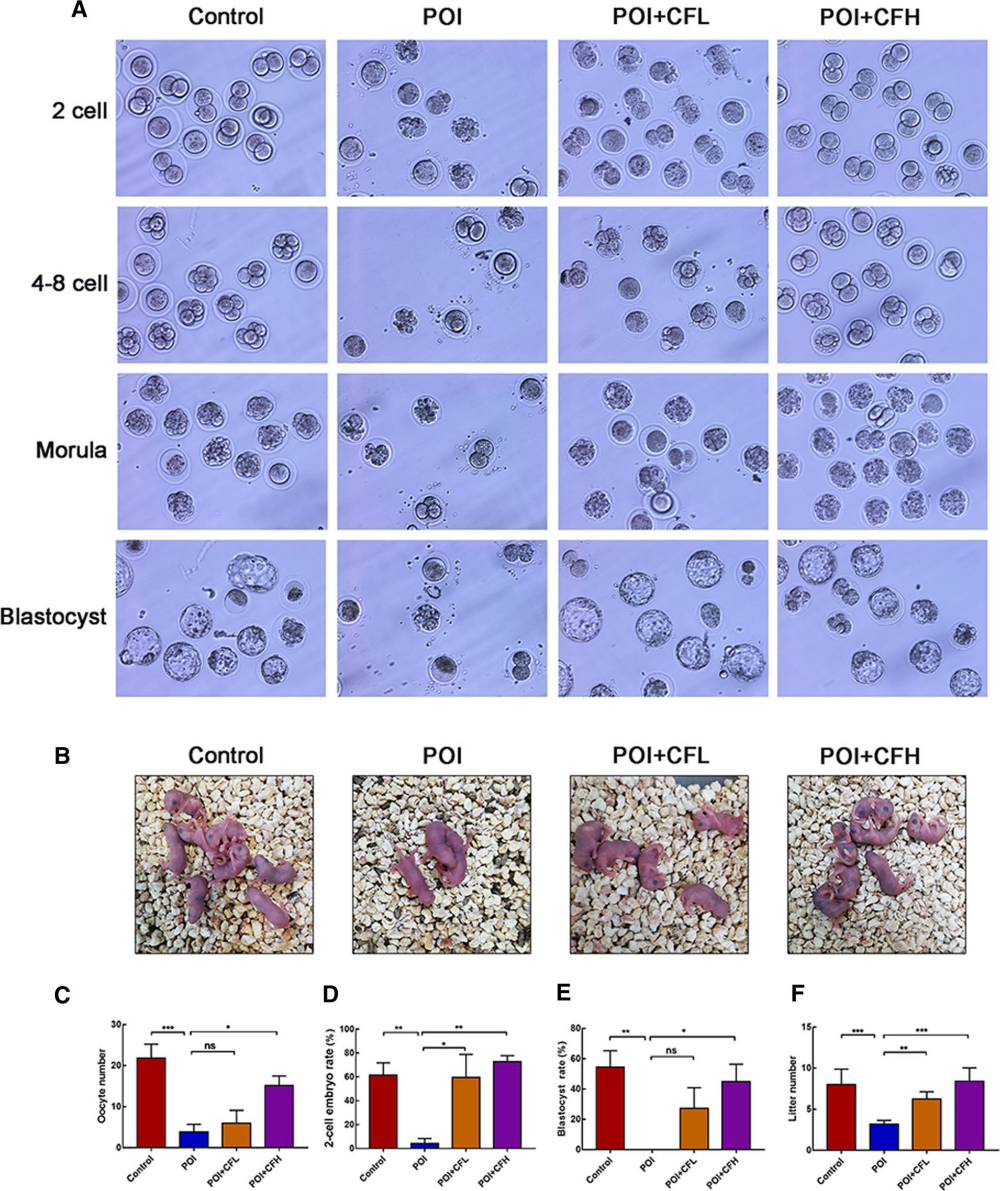
CEFFE improved the fertility of POI mice.** A** Representative images of 2-cell embryo, 4–8-cell embryo, morular and blastocyst from the different groups. **B** Typical images of progeny in each group. **C** Statistical analysis of oocytes retrieved in each group (*n* = 6). **D** Statistical analysis of the 2-cell embryo ratios (2-cell embryo/total oocytes retrieved) in each group (*n* = 6). **E** Statistical analysis of blastocyst ratios (blastocyst/2-cell embryo) in each group (*n* = 6). **F** Statistical analysis of litter size in each group (*n* = 5–8). All data are represented as the mean ± SD. ns: *P* > 0.05, *P < 0.05, **P < 0.01, ***P < 0.001, compared with POI group

Previous experiments demonstrated that CEFFE could rescue sex hormones and promote the growth of follicles in POI mice. Next, we further examined the fertility of mice. First, oocytes were collected and cultured in vitro (Fig. 2A). The number of retrieved oocytes was much lower in the POI group than in the control group. After treatment with CEFFE, the retrieved oocytes increased obviously in the POI + CFH group, but there was no difference in the POI + CFL group compared to the POI group (Fig. 2C). In addition, similar results in the ratios of 2-cell embryos and blastocysts were observed (Fig. 2D, E). These results indicated that CEFFE could promote the development of embryos. Next, we found that the litter sizes of POI mice were smaller than those in the control group, were slightly increased in the POI + CFL group and were significantly increased in the POI + CFH group, as shown by the mating test (Fig. 2B, F; Additional file 2: Fig. S6). These data showed the effect of CEFFE on the fertility of POI mice based on the number of oocytes retrieved, embryo development and offspring and indicated that CEFFE could restore the impaired fertility in POI mice, especially in the POI + CFH group.

### Safety evaluation of mice that received CEFFE treatment

The cancer markers PTEN and P53 were examined to assess the ovarian oncogenicity of CEFFE, and the results demonstrated that CEFFE had no side effects that led to tumorigenesis (Additional file 2: Fig. S2AB). To explore the safety of CEFFE in parent mice, the organ index in each group was evaluated at 8 weeks after treatment with CEFFE. The data showed that the organ indices of the heart, liver, spleen, lung, kidney and uterus were not different among the groups (Additional file 2: Fig. S2CD). To assess the safety of CEFFE on the offspring in the POI + CFL and POI + CFH groups, which were pretreated with CEFFE. The body weights of the pups were monitored weekly until 8 weeks, and then the organ index was assessed. Similar results were observed; no differences were observed in the organ indices of heart, liver, spleen, lung, kidney and testis or uterus and ovary, suggesting that CEFFE had no adverse effects on the growth and biosafety of the offspring (Additional file 2: Fig. S3).

### CEFFE increased angiogenesis in the ovarian medulla, increased proliferation and decreased GC apoptosis in follicles

**Fig. 3:**
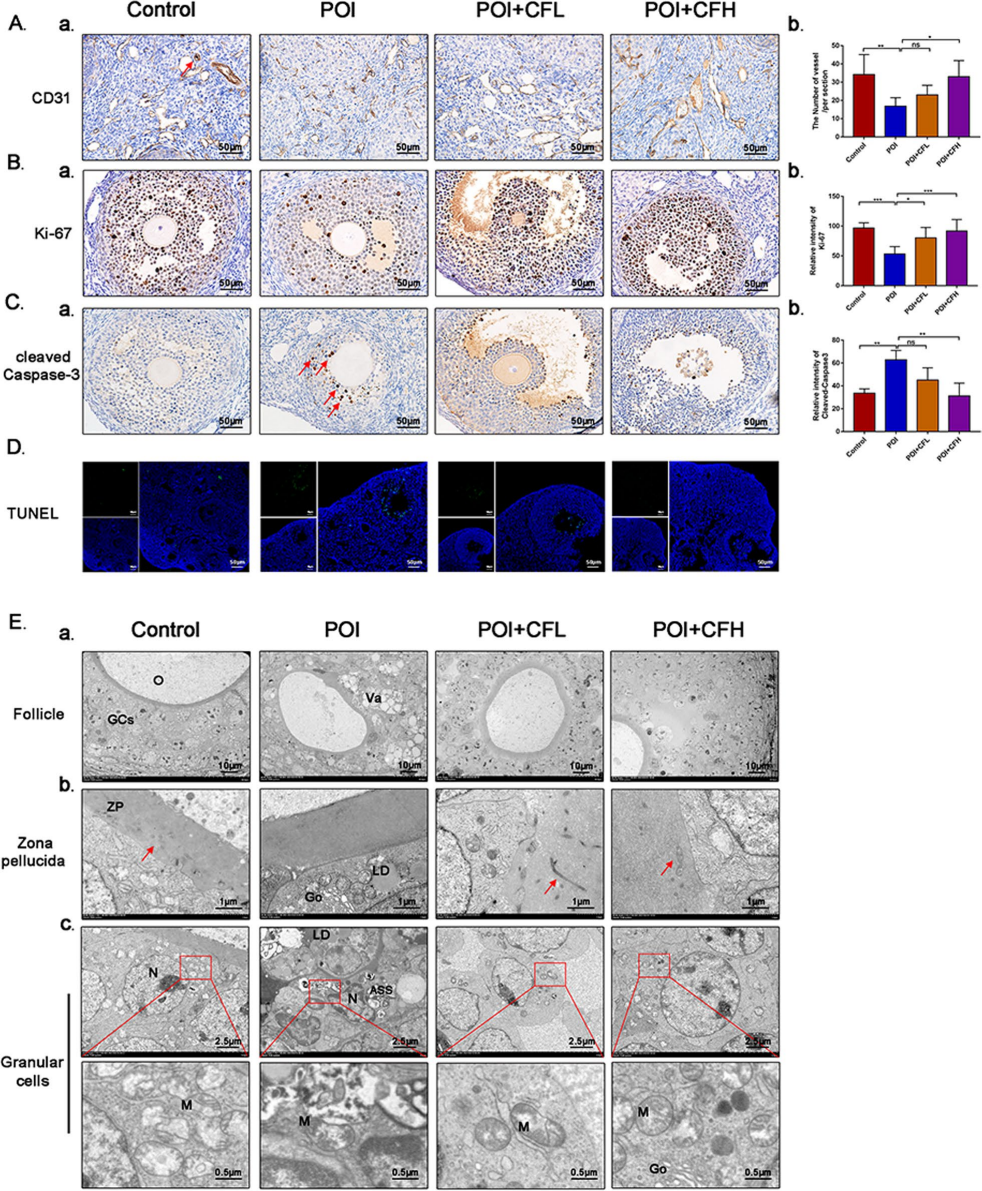
Evaluation of the role of CEFFE in protecting ovarian function at the tissue level. **A** Representative micrographs of vessels in the medulla that were stained for CD31 (**a**) and quantitative vessel numbers in the different groups (**b**), (*n* = 6). The red arrows indicate blood vessels. Scale bar = 50 μm. **B** Representative micrographs showing follicles stained for Ki-67 (**a**) and quantitative analyses (**b**), (*n* = 6). Scale bar = 50 μm. **C** Representative micrographs of follicles stained for Cleaved-Caspase3 (**a**) and quantitative analysis (**b**) (*n* = 6). The red arrows indicate apoptotic cells. Scale bar = 50 μm. **D** GC apoptosis was measured by the TUNEL apoptosis test. Green fluorescence represents apoptotic cells, and blue fluorescence represents the nuclei of all cells. Scale bar = 100 μm. **E** TEM observations of ultrastructural changes in the ovary. **a** The ultrastructure of follicles. Scale bar = 10 μm. **b** The ultrastructure of the zona pellucida. The red arrow shows the material in the zona pellucida. Scale bar = 1 μm. **c** The ultrastructure of GCs. Scale bar = 2.5 μm or 0.5 μm. O: Oocyte, GCs: granulosa cells, Va: Vacuoles, ZP: Zona pellucida, LD: Liquid droplet, Go: Golgi complex, N: Nucleus, Ly: Lysosome, ASS: Autolysosome, M: mitochondria. Data are represented as the mean ± SD. ns: *P* > 0.05, *P < 0.05, **P < 0.01, ***P < 0.001, compared with POI group

Based on the efficacy and safety of CEFFE on improving reproduction in POI mice, we next explored the effect of CEFFE on ovaries at the tissue level, including the medulla and cortex. The medulla is mainly composed of blood vessels and lymphatic vessels. Angiogenesis in the medulla was observed by assessing CD31 (a marker of blood vessels). There was reduced angiogenesis in the POI group, while increased angiogenesis occurred in the POI + CFL and POI + CFH groups (Fig. 3A). In addition, the ovarian cortex is composed of follicles with different morphologies, and follicles are mainly composed of oocytes and GCs (GCs). The proliferation and apoptosis of cells in ovary was detected with Ki67 (a proliferation marker protein) and Cleaved-Caspase3 (an apoptotic marker protein) by IHC, respectively. The results revealed more Ki67-positive and fewer Cleaved-Caspase3-positive GCs in the POI + CFL and POI + CFH groups than in the POI group (Fig. 3B, C). Subsequently, to further confirm the anti-apoptotic effect of CEFFE on GCs, TUNEL staining showed increased apoptotic cells, which were located in the GC layer, in the POI group, and reduced apoptotic cells in the POI + CFL and POI + CFH groups (Fig. 3D). Next, to observe the change of microscopic morphology in ovaries, TEM observation was performed. Significantly, in the POI group, we found an increase in abnormally disordered GCs in follicles and less material composition of exchange in the zona pellucida, which is typically abundant between GCs and oocytes and provides essential nutrients for oocytes (Fig. 3E-ab). Moreover, more vacuoles and degenerated mitochondria with abnormal, round shapes and fewer cristae were observed in the GCs of POI mice, suggesting severe mitochondrial damage. However, in the POI + CFL and POI + CFH groups, CEFFE significantly attenuated this damage, and an increased number of GCs with regular arrangements and numerous spherical mitochondria with obvious cristae in their matrix were observed (Fig. 3E-c). Taken together, these results suggest that CEFFE could suppress GCs apoptosis, contributing to a reduction in atretic follicles and an increase in healthy follicles.

### CEFFE promoted CTX-damaged KGN cell proliferation in vitro

**Fig. 4:**
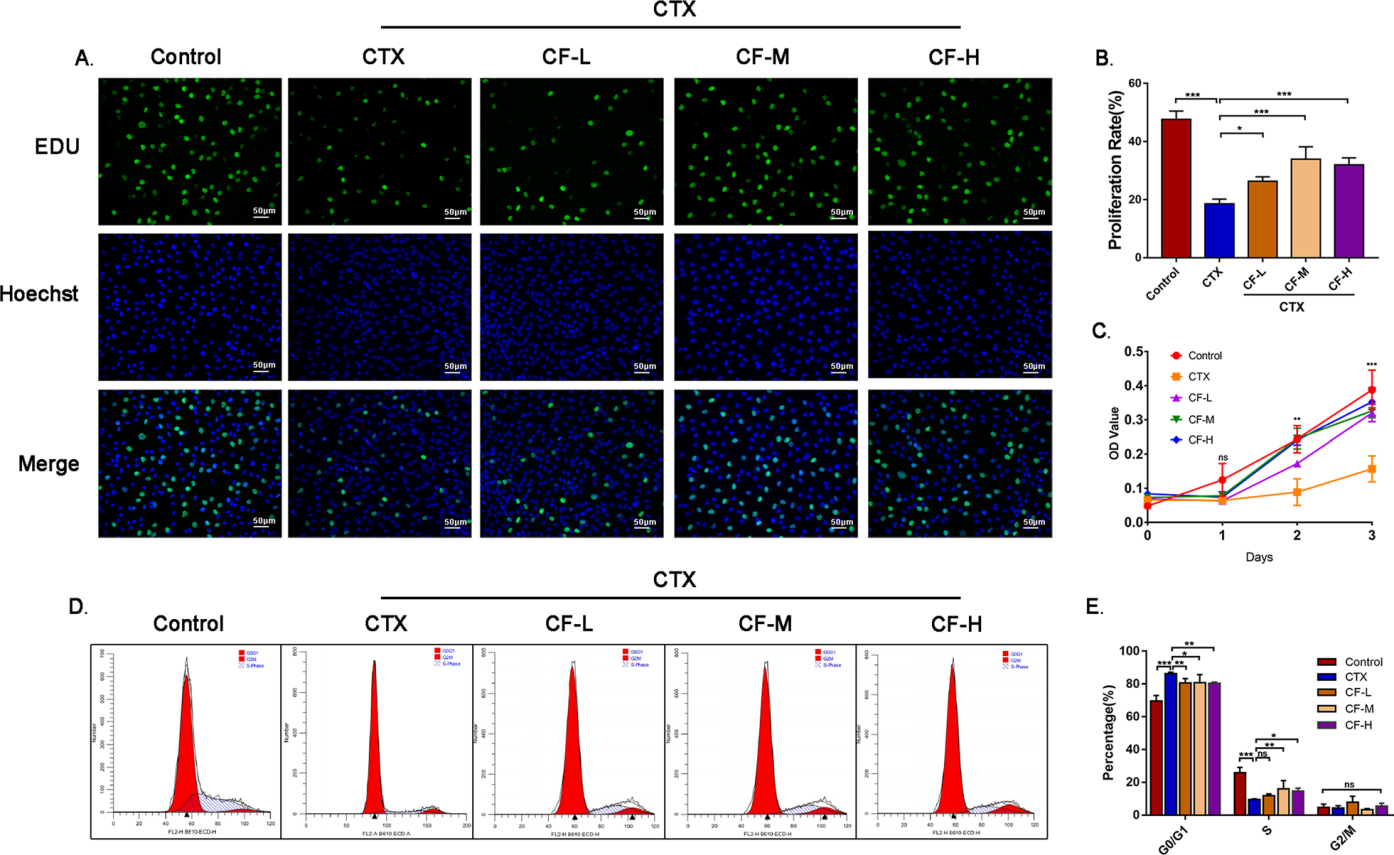
CEFFE contributed to the proliferation of CTX-damaged KGN cells. **A** Cell proliferation was measured by EdU assays. Green fluorescence represents proliferative cells, and blue fluorescence represents the nuclei of all cells. Scale bar = 50 μm. **B** Semiquantitative results of the EdU assay. **C** Cell proliferation was measured by CCK-8 assays. **D** The cell cycle was detected by flow cytometry. **E** Quantitative analysis of the cell cycle distributions of G0/G1, S, and G2/M phases. CF-L: CEFFE at a concentration of 0.06 µg/µL, CF-M: CEFFE at a concentration of 0.15 µg/µL, CF-H: CEFFE at a concentration of 0.3 µg/µL. The data are presented as the mean ± SD. ns: *P* > 0.05, *P < 0.05, **P < 0.01, ***P < 0.001, compared with CTX group

The KGN cell line was used as an in vitro model to investigate the effect of CEFFE on human ovarian granulosa cells. First, we used several concentrations of CTX (0 μM, 50 μM, 100 μM, 200 μM, 250 μM and 300 μM) and examined the viability of KGN cells, and the results showed that viability decreased in a concentration- and time-dependent manner. At 48 h after exposure to 250 μM CTX, cell viability declined by 50%, and this concentration was used to establish CTX-damaged cells in vitro (Additional file 2: Fig. S4). To further explore the effect of CEFFE on KGN cell proliferation, we cultured CTX-damaged KGN cells with three different concentrations of CEFFE (CF-L: 0.06 µg/µL, CF-M: 0.15 µg/µL, CF-H: 0.3 µg/µL) for 48 h and then performed EdU proliferation assays. The results indicated an increase in EdU-positive cells in the CEFFE groups, which was opposite of the changes induced in the CTX group, especially in the CF-M and CF-H groups (Fig. 4A, B). Similar results were observed in the CCK-8 test, which extended the CEFFE intervention time to 72 h (Fig. 4C). Moreover, the cell cycle profiles suggested that there were fewer cells in S phase (the stage of DNA synthesis) in the CTX-damaged group than in the other groups. After treatment with CEFFE, more cells entered S phase, and fewer cells entered G0/G1 phase (Fig. 4D, E). These results indicated that CEFFE restored the proliferation of CTX-damaged KGN cells, which could contribute to the development of follicles in vivo.

### CEFFE inhibited CTX-induced KGN cell apoptosis and improved mitochondrial function

**Fig. 5:**
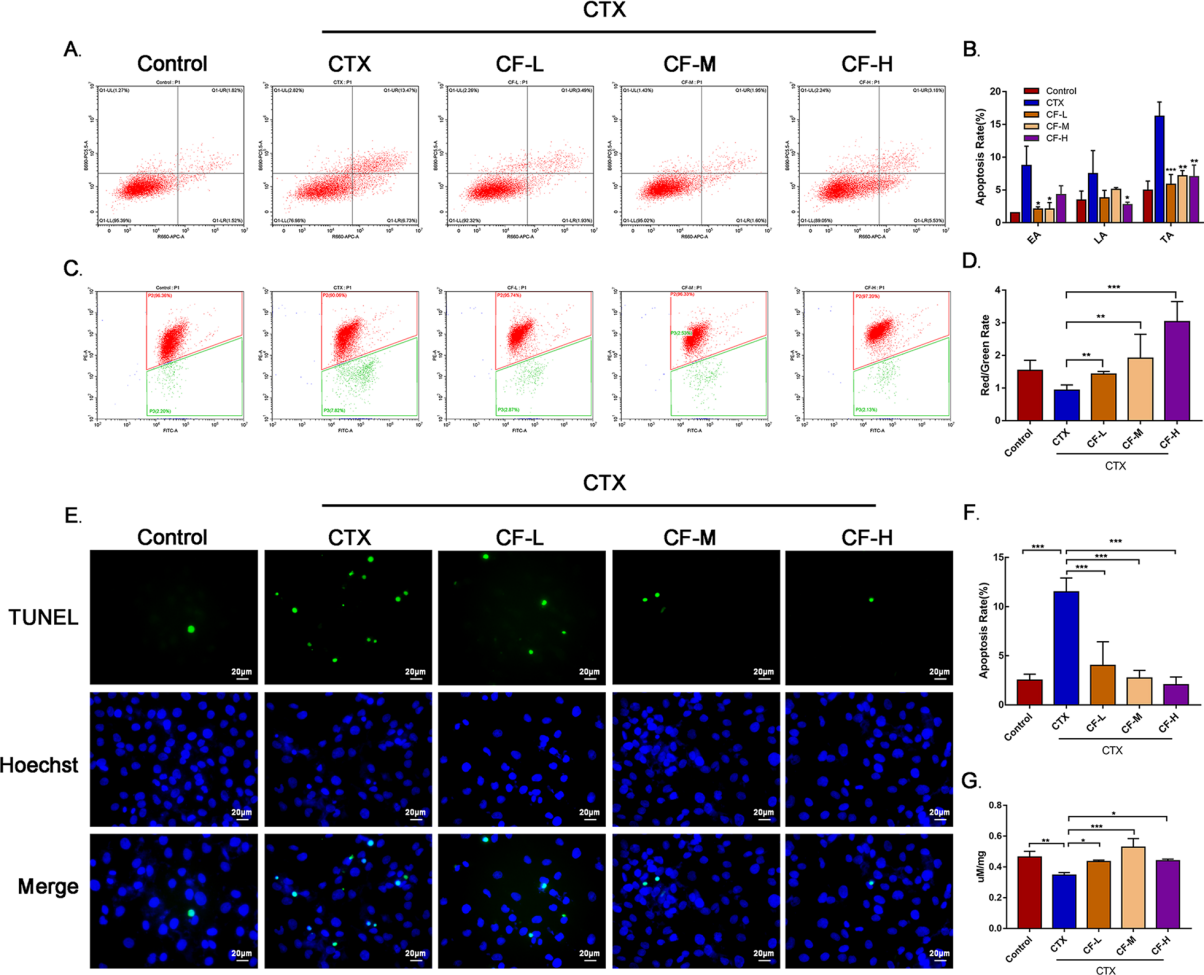
CEFFE inhibited apoptosis in CTX-damaged KGN cells. **A** KGN cell apoptosis was measured by Annexin V APC/7-AAD and flow cytometry. **B** Quantitative analysis of apoptosis, as determined by the Annexin V APC/7-AAD test. **C** Mitochondrial membrane potential was detected by JC-1 and flow cytometry. Red indicates intact mitochondrial membrane potential; green indicates damaged mitochondrial membrane potential. **D** Quantitative analysis of the fluorescence intensity of JC-1 (the rate of red/green). **E** KGN cell apoptosis was measured by TUNEL assays. Green fluorescence represents apoptotic cells, and blue fluorescence represents the nuclei of all cells. Scale bar = 20 μm. **F** Quantitative analysis of the TUNEL assay results. **G** Effect of CEFFE on the ATP levels in KGN cells. The data are represented as the mean ± SD. *P < 0.05, **P < 0.01, ***P < 0.001, compared with CTX group

To explore whether CEFFE could inhibit apoptosis in CTX-damaged KGN cells, an Annexin V APC/7-AAD apoptosis test was performed, and the results indicated that CF-L and CF-M could reduce early apoptosis, while CF-H could reduce late apoptosis in KGN cells (Fig. 5A, B). As indicated by TEM, mitochondrial damage was the most obvious of all disordered organelles in the GCs of POI mice, and this effect was closely related to early apoptosis in cells. Thus, early apoptosis in KGN cells was measured by measuring mitochondrial membrane potential with JC-1 and flow cytometry (Fig. 5C, D). The ATP level was also assayed (Fig. 5G). These results revealed that CEFFE could rescue mitochondrial membrane potential and ATP levels in CTX-damaged KGN cells, indicating that CEFFE promoted the recovery of mitochondrial function and inhibited early apoptosis. Furthermore, a TUNEL assay was carried out to detect late apoptosis, and the results showed much more apoptotic cells in the CTX group and a dose-dependent decrease in positive cells in the CF-L, CF-M and CF-H groups (Fig. 5E, F). Taken together, these results suggested that CTX could lead to apoptosis and that CEFFE exerted an anti-apoptotic effect on CTX-damaged KGN cells.

### Effect of CEFFE on CTX-damaged hGCs

**Fig. 6:**
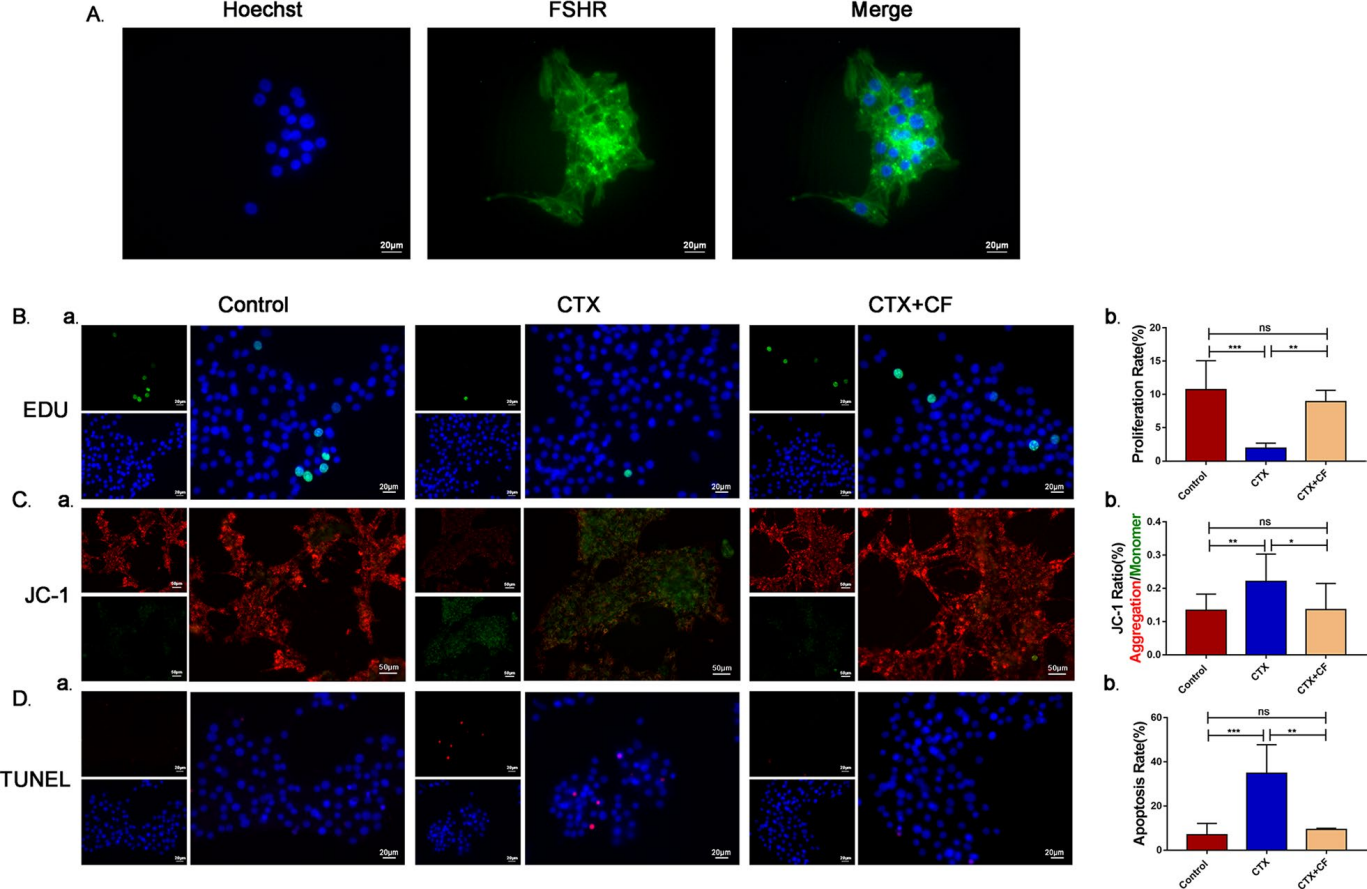
Effect of CEFFE on hGCs **A** The identification of hGCs by FSHR expression. FSHR was labeled with green fluorescence, and the nucleus was labeled with blue fluorescence. Scale bar = 20 μm. **B** EdU assays were used to monitor cell proliferation (**a**) and quantified (**b**) (*n* = 4). Green fluorescence represents proliferative cells, and blue fluorescence represents the nuclei of all cells. Scale bar = 20 μm. **C** JC-1 fluorescence assays were used to monitor mitochondrial membrane potential (**a**) and quantified (**b**) (*n* = 4). Red indicates intact mitochondrial membrane potential, and green indicates damaged mitochondrial membrane potential. Scale bar = 50 μm. **D** TUNEL assays were used to monitor cell apoptosis (**a**) and quantified (**b**) (*n* = 3). Red fluorescence represented the apoptotic cells, and blue fluorescence represented the nucleus of all cells. Scale bar = 20 μm. Data are represented as the mean ± SD. ns: *P* > 0.05, compared with Control group; *P < 0.05, **P < 0.01, ***P < 0.001, compared with CTX group

We showed that CEFFE could promote proliferation, rescue mitochondrial function and inhibit apoptosis in CTX-damaged KGN cells. To further confirm the effect of CEFFE, hGCs were extracted and identified by the protein expression of FSHR, which is specifically located in the cytoplasm of hGCs (Fig. 6A). The results showed that nearly all cells expressed FSHR, indicating that the cells were hGCs. Then, hGCs were damages with 250 μM CTX, and only the medium concentration of CEFFE (CTX + CF) was tested. The EdU results showed that CEFFE could promote the proliferation of hGCs (Fig. 6B), the JC-1 assay results showed the rescue of mitochondrial function and inhibition of early apoptosis (Fig. 6C), and the TUNEL assay results shows the inhibition of late apoptosis in hGCs (Fig. 6D). These results indicated that CEFFE could play a similar role in KGN cells and hGCs.

### RNA-seq analysis of the mouse ovarian GCs transcriptome treated with CEFFE

**Fig. 7:**
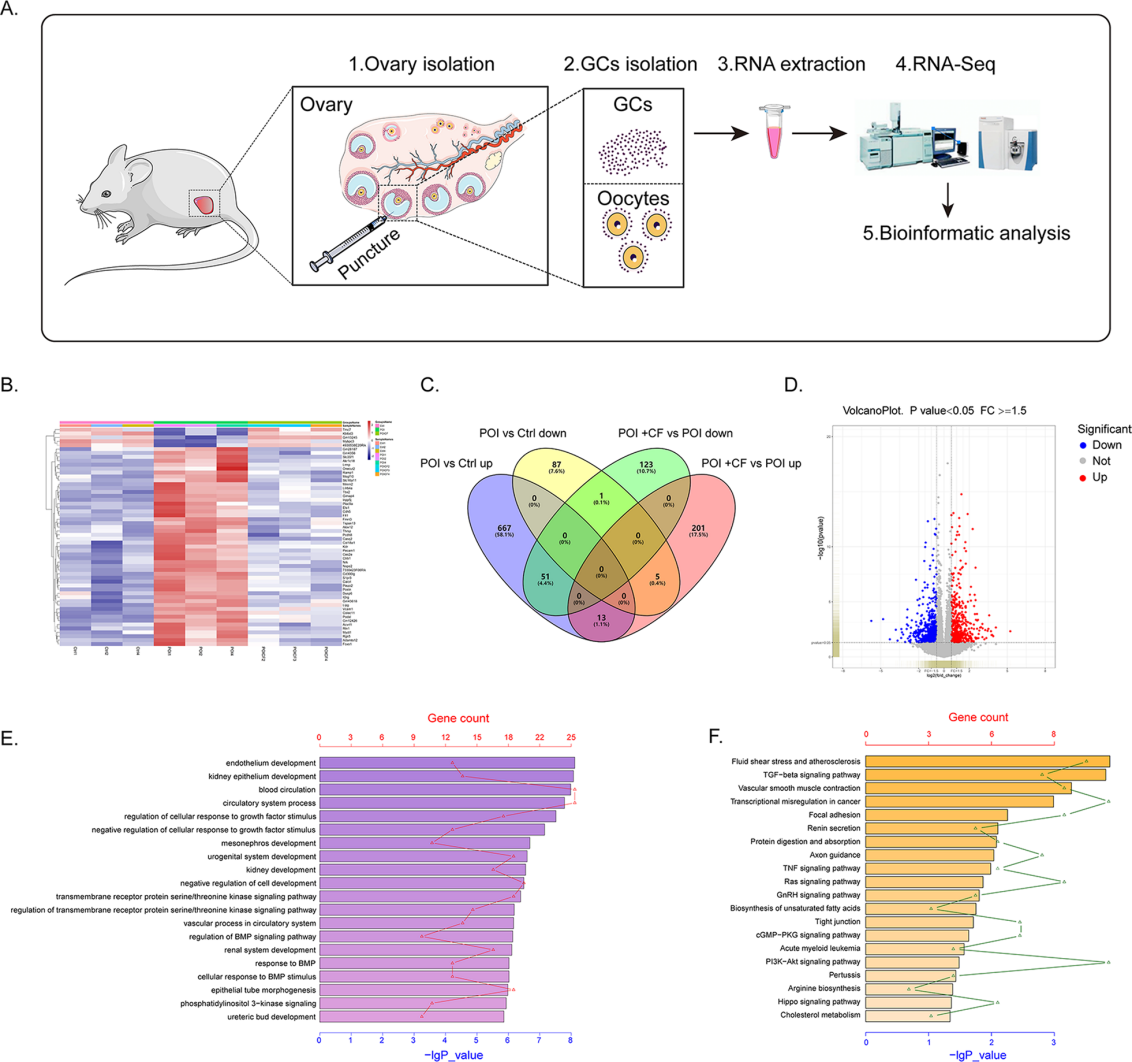
The mRNA-Seq analysis of mouse ovarian GCs. **A** Schematic design of the mRNA-Seq analysis of mouse ovarian GCs. POI: POI mice treated with CTX and BUS; POI + CF: POI mice treated with CTX and BUS, followed by high-dose CEFFE (3 µg/µl, 200 µl) treatment. **B** The heatmap showing DEGs in the control, POI and POI + CF groups with an absolute fold change > 1.5 and P value < 0.05. (*n* = 3). **C** Venn diagram showing gene counts in the POI versus control and POI + CF versus POI groups. **D** Volcano plot showing DEGs in the POI + CF group compared to the POI group. Among the 394 genes with an absolute fold change > 1.5, 219 genes were upregulated, while 175 genes were downregulated. **E** Top 20 GO terms for DEGs. **F** Top 20 KEGG terms for DEGs

Next, we explored how CEFFE exerts its anti-apoptotic effect on GCs. We performed RNA sequencing (RNA-seq) of ovarian GCs derived from the control group, POI group and POI + CF group (only the POI + CFH group was chosen) (Fig. 7A). The scatter diagram demonstrated that a total of 394 genes had an absolute fold change greater than 1.5; among them, 219 genes were upregulated, while 175 genes were downregulated in the GCs in the POI + CF group compared with the POI group (Fig. 7B–D). Then, we used GO and KEGG analyses to determine the roles of the altered pathways in GCs. The data showed that “regulation of cellular response to growth factor stimulus”, “transmembrane receptor protein serine/threonine kinase signaling pathway” and “cellular response to BMP stimulus” were enriched among the top 20 GO terms, which were closely related to cytokine interactions with cells, according to the fact that there were various cytokines in CEFFE (Fig. 7E). Moreover, KEGG pathway analysis revealed that the TGFβ, TNF, Ras, PI3K-Akt and Hippo signaling pathways were all enriched (Fig. 7F). As previous reported, those pathways are essential for the function of GCs and folliculosgenesis which contribute to the improvement of ovarian function.

Besides, our previous study demonstrated that CEFFE contained 1767 active proteins and possesses strong pro-proliferation and anti-apoptosis activity. There are 1693 proteins was homologous between human and mouse (Additional file 4: Table S2). Among them, 131 proteins were related to cell proliferation and 141 proteins were related to cell apoptosis (Additional files 5, 6: Tables S3, S4). We speculated that those proteins possibly contribute to the process of pro-proliferation and anti-apoptosis of granulosa cells which were crucial to the improvement of ovarian function.

## Discussion

POI, a main cause of infertility in reproductive women, is a challenging and troublesome problem associated with ART [22]. Currently, there is a lack of effective treatments to improve the fertility of women with POI in the clinic [23, 24]. In this study, we first showed that CEFFE restored ovarian function and rescued fertility in POI mice established by chemotherapeutic drugs. Meanwhile, we verified CEFFE could increase proliferation and inhibit apoptosis of human ovarian granulosa cells, which may provide a new clinical strategy for the treatment of POI.

As a vital gonadal organ, the ovary produces sex hormones and facilitates reproduction by producing oocytes for fertilization [25], which could affect female fertility and even overall lifespan [26, 27]. In this study, after treatment with CEFFE, ovarian size, estrus cycle and sex hormones (AMH, E_2_ and FSH) in POI mice were recovered to normal levels, suggesting that CEFFE could improve the secretory function of the ovary (Fig. 1). Considering the difficulties of current clinical treatment, reproductive repair is highly noteworthy. Therefore, we further evaluated the effect of CEFFE on fertility. In the POI + CFL and POI + CFH groups, increased numbers of growing follicles and reduced numbers of atretic follicles were observed in the ovary. Next, embryonic culture experiments showed that the quantity and quality of oocytes were obviously improved. Importantly, the mating results showed that the litter sizes of POI mice were significantly reduced and were dramatically increased after CEFFE treatment (Fig. 2). Thus far, we comprehensively demonstrated that CEFFE could restore the fertility of POI mice based on the number of follicles and oocytes retrieved, embryonic development and even final litter size. Moreover, as this treatment involves stem cells, it is necessary to evaluate the potential risks of tumorigenicity and gene integration mutations on organisms when researching biologically active agents [28]. Thus, we evaluated tumor risk by measuring tumor markers (PTEN and P53) in the ovary. Moreover, CEFFE was shown to have no adverse effects on the growth of the parents or offspring, as indicated by the body weight and organ index (Additional file 2: Fig. S2–S3). Collectively, these animal experiments fully verified the efficacy and safety of CEFFE in repairing ovarian secretion and reproductive function.

Based on the efficacy and safety of CEFFE for POI treatment, we further explored the effect of CEFFE on GCs function. Ovarian reserve function is determined by the quantity and quality of oocytes, and GCs can provide necessary nutrients for oocyte maturation; the status of GCs determines the fate of oocytes. Proliferative GCs promote follicular maturation and ovulation, and apoptotic GCs lead to follicular degradation and atresia, which is the main mechanism of follicular atresia [29–31] and is closely related to the occurrence of POI and embryonic development [32, 33]. Therefore, exploring the effect of CEFFE on GCs is beneficial for clarifying the mechanism by which CEFFE can treat POI. Ovarian tissue sections showed that CEFFE promoted GCs proliferation, inhibited apoptosis, and changed mitochondrial morphology (Fig. 3). Next, three different concentrations of CEFFE were cultured with CTX-injured KGN cells, and the results suggested that CEFFE promoted KGN cell proliferation, improved mitochondrial function, and inhibited apoptosis (Figs. 4, 5). The middle concentration was the most effective. GCs are divided into peripheral GCs and cumulus GCs, which closely surround oocytes and are more important for oocyte maturation than peripheral GCs [34]. To our knowledge, this is the first study to focus on the effects of CTX and CEFFE on cumulus GCs, and similar results were observed when CTX-injured hGCs were treated with a moderate concentration of CEFFE (Fig. 6). Moreover, the effect of CEFFE on proliferation was reported in a study of skin repair, and an anti-apoptotic effect was reported in osteoporosis, which was consistent with our findings [18, 35]. In addition to its effect on proliferation and apoptosis, CEFFE can also enhance the mitochondrial function of GCs. It has been reported that CTX upregulates the apoptotic protein Bax, which is located in the cytoplasm and intercalates into the mitochondrial outer membrane, disrupting the mitochondrial membrane potential and activating the apoptotic cascade. Thus, we proposed that CEFFE could inhibit GCs apoptosis and promote proliferation by inhibiting the apoptotic cascade triggered by CTX-impaired mitochondrial function. In turn, this treatment improves granulosa cell function and affects ovarian function.

Above results showed that CEFFE inhibited apoptosis and promoted the proliferation of GCs in ovarian tissue and cell experiments (KGN cell line and hGCs), respectively. We further investigated the mechanism by which CEFFE affected the function of GCs by comparing the DEGs in ovarian GCs from POI + CFH and POI mice based on the mRNA transcriptome and KEGG analysis. The results suggested many crucial signaling pathways were regulated by CEFFE. For example, TGFβ pathway is involved in early follicle growth and GCs differentiation, which are closely related to the occurrence of ovarian dysfunction [36–38]. PI3K-Akt pathway is related to manipulate the dormancy and activation of primordial follicles [39]. Hippo pathway also regulates [40] the follicle survival and granulosa cells proliferation which is critical in folliculosgenesis.

Next, combined the proteins in CEFFE, we further discuss the underlying regulation mechanism between CEFFE and ovary function. On one hand, there were 131 and 141 proteins in CEFFE which related to cell proliferation and apoptosis, respectively. On the other hand, previous studies have shown that CEFFE is abundant in growth factors, such as GDNF, TGFβ, and BDNF [15]. Both of them play central roles in regulating folliculogenesis in the ovary [41–43]. However, which and how the specific proteins regulate the vital pathway need to be further explored. For example, TGFβ, the second most abundant growth factor in CEFFE, as reported before, can directly activate the TGFβ pathway, as well as promote cell proliferation and antagonize apoptosis [44, 45], which might play a vital role on the recovery of ovarian function.

## Conclusions

Thus far, we found that CEFFE could restore ovarian function and rescue the fertility of POI mice possibly by antagonizing CTX-induced GCs apoptosis, providing a novel therapeutic strategy to ameliorate POI and improve female reproductive health. In addition, more research should be conducted to identify the specific functional components involved in ovary repair and how they work, such as by local positioning or systemically, which would provide a new research direction for the precise treatment of POI.

## Supplementary Information

**Additional file 1: Methods.** Establish the POI mice model by different does.

**Additional file 2: Fig. S1.** Establishment of POI model in mice by chemotherapeutic agents. **Fig. S2.** Biosafety assessment of CEFFE on parent mice. **Fig. S3.** Biosafety assessment of CEFFE on pups. **Fig. S4.** Establishment of CTX-damaged KGN cell model. **Fig. S5.** All the gross morphology of ovaries from different groups with four individual experiments. **Fig. S6.** The original images of pups from different groups.

**Additional file 3: Table S1.** The detail information of antibodies used in this study.

**Additional file 4: Table S2.** Homologene proteins human to mouse in CEFFE.

**Additional file 5: Table S3.** Apoptosis related proteins in CEFFE.

**Additional file 6: Table S4.** Proliferation related proteins in CEFFE.

## Data Availability

The datasets used during the current study are available from the corresponding author on reasonable request.

## Abbreviations

CEFFE: Cell-free fat extract
POI: Premature ovarian insufficiency
E_2_: Estrogen
FSH: Follicle stimulating hormone
ART: Assisted reproductive technology
ADSCs: Adipose-derived mesenchymal stem cells
CTX: Cyclophosphamide
BUS: Busulfan
ELISA: Enzyme-linked immunosorbent assay
AMH: Anti-Mullerian hormone
PMSG: Pregnant mare serum gonadotropin
hCG: Human chorionic gonadotropin
IHC: Immunohistochemistry
TEM: Transmission electron microscopy
hGCs: Human primary ovarian cumulus granulosa cells
KGN: Human ovarian granulosa-like tumor cell line
CCK-8: Cell counting kit
OD: Optical density
GCs: Granulosa cells
RNA-Seq: RNA sequencing
GO: Gene ontology
KEGG: Kyoto encyclopedia of genes and genomes
DEGs: Differentially expressed genes
