# Supplementary material for: Cell-free fat extract improves ovarian function and fertility in mice with premature ovarian insufficiency
Source: Stem Cell Res Ther. 2022 Jul 16;13:320. doi: 10.1186/s13287-022-03012-w (PMC9288692; doi:10.1186/s13287-022-03012-w)
Supplement: Supplementary file 1 — Additional file 1: Methods. Establish the POI mice model by different does. [file 13287_2022_3012_MOESM1_ESM.docx]

**Cell-free Fat Extract Improves Ovarian Function and Fertility in Mice with Premature Ovarian Insufficiency**

**Additional file 1**

**Supplementary Methods：**

**Establish the POI mice model by different does**

Firstly, four different doses of Cyclophosphamide (CTX, Sigma-Aldrich) and Busulfan (BUS, Sigma-Aldrich) was used to explore the optimal concentration for POI mice model establishment. The female mice were divided into five groups randomly, receiving a single intraperitoneal injection with different does of CTX and BUS except control group: Group 1 was conducted with of 80 mg /kg CTX and 12 mg/kg BUS. Group 2 was conducted with 100 mg/kg CTX and 12 mg/kg BUS. Group 3 was conducted with 120 mg/kg CTX and 12 mg/kg BUS. Group 4 was conducted with of 120 mg/kg CTX and 30mg/kg BUS. After two weeks, the serum, ovaries were collected for further identifying the feasibility of POI mice model.
