# Supplementary material for: Cell-free fat extract improves ovarian function and fertility in mice with premature ovarian insufficiency
Source: Stem Cell Res Ther. 2022 Jul 16;13:320. doi: 10.1186/s13287-022-03012-w (PMC9288692; doi:10.1186/s13287-022-03012-w)
Supplement: Supplementary file 2 — Additional file 2: Fig. S1. Establishment of POI model in mice by chemotherapeutic agents. Fig. S2. Biosafety assessment of CEFFE on parent mice. Fig. S3. Biosafety assessment of CEFFE on pups. Fig. S4. Establishment of CTX-damaged KGN cell model. Fig. S5. All the gross morphology of ovaries from different groups with four individual experiments. Fig. S6. The original images of pups from different groups. [file 13287_2022_3012_MOESM2_ESM.docx]

**Cell-free Fat Extract Improves Ovarian Function and Fertility in Mice with Premature Ovarian Insufficiency**

**Additional file 2**

**Supplementary Figures**

**
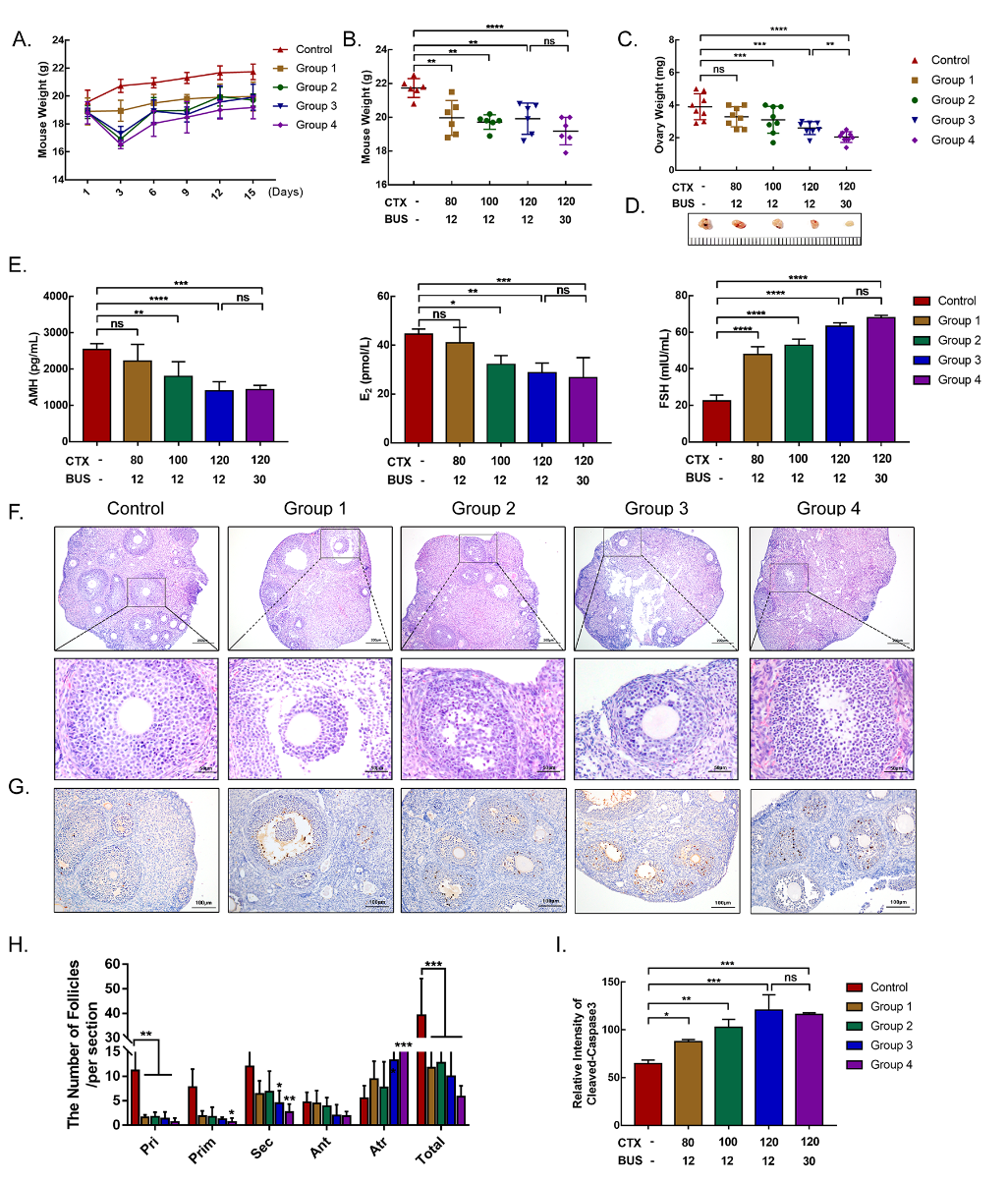
**

**Supplementary Figure S1. Establishment of POI model in mice by chemotherapeutic agents.**

**(A)** The body weight of mice was monitored for two weeks after injected with CTX and BUS (n=6). (**B)** The body weight of mice was analyzed at the last day (n=6). (**C)** The ovary weight of mice was measured at 2 weeks after modeling (n=9). **(D)** The representative morphology of ovaries from each group. **(E)** The content of AMH, E_2_ and FSH in serum was measured by ELISA (n=5). **(F)** The ovaries were stained with H&E. Scale bars=200μm or 50μm. **(G)** Cleaved-Caspase3 antibody was applied in IHC method to manifest the apoptotic granulosa cells in ovary (n=3), Scale bar =100μm. **(H)** The number of different follicles was counted (n=5-7). Pri: Primordial follicles, Prim: Primary follicles, Sec: Secondary follicles, Ant: Antral follicles, Atr: Atretic follicles, Total: The total number of health follicles (including primordial, primary, secondary and antral follicles). **(I)** The quantitative statistics for Cleaved-Caspase3. Data are represented as the mean ± SD, ns: *P*>0.05, ^*^*P* < 0.05, ^**^*P* < 0.01,^***^*P* < 0.001.

**
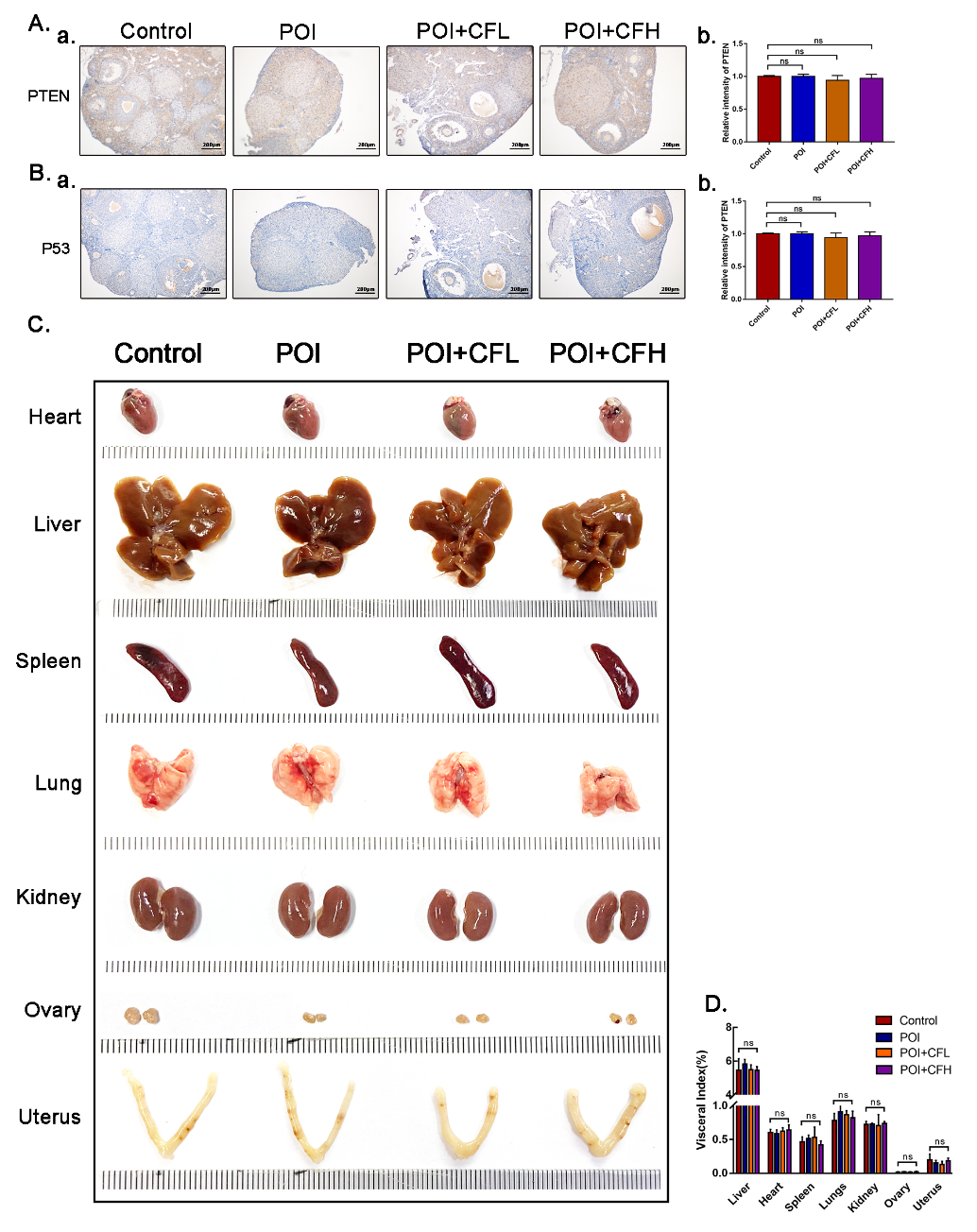
**

**Supplementary Figure S2. Biosafety assessment of CEFFE on parent mice.**

**(A)** Expression of PTEN as an indication of suppressive gene was measured by IHC images (a) with quantitative analysis (b). Scale bars=200μm. (n=3-4). (**B).** Expression of P53 as an indication of oncogenic gene was measured by IHC images (a) with quantitative analysis (b). Scale bars=200μm. (n=3-4). **(C)** The morphology of heart, live, spleen, lung, kidney, ovary and uterus from each group at 8w after treated with CEFFE. **(D)** The organ index of parent mice (n=5). Data are represented as the mean ± SD. ns: *P*>0.05, no statistical difference.

**
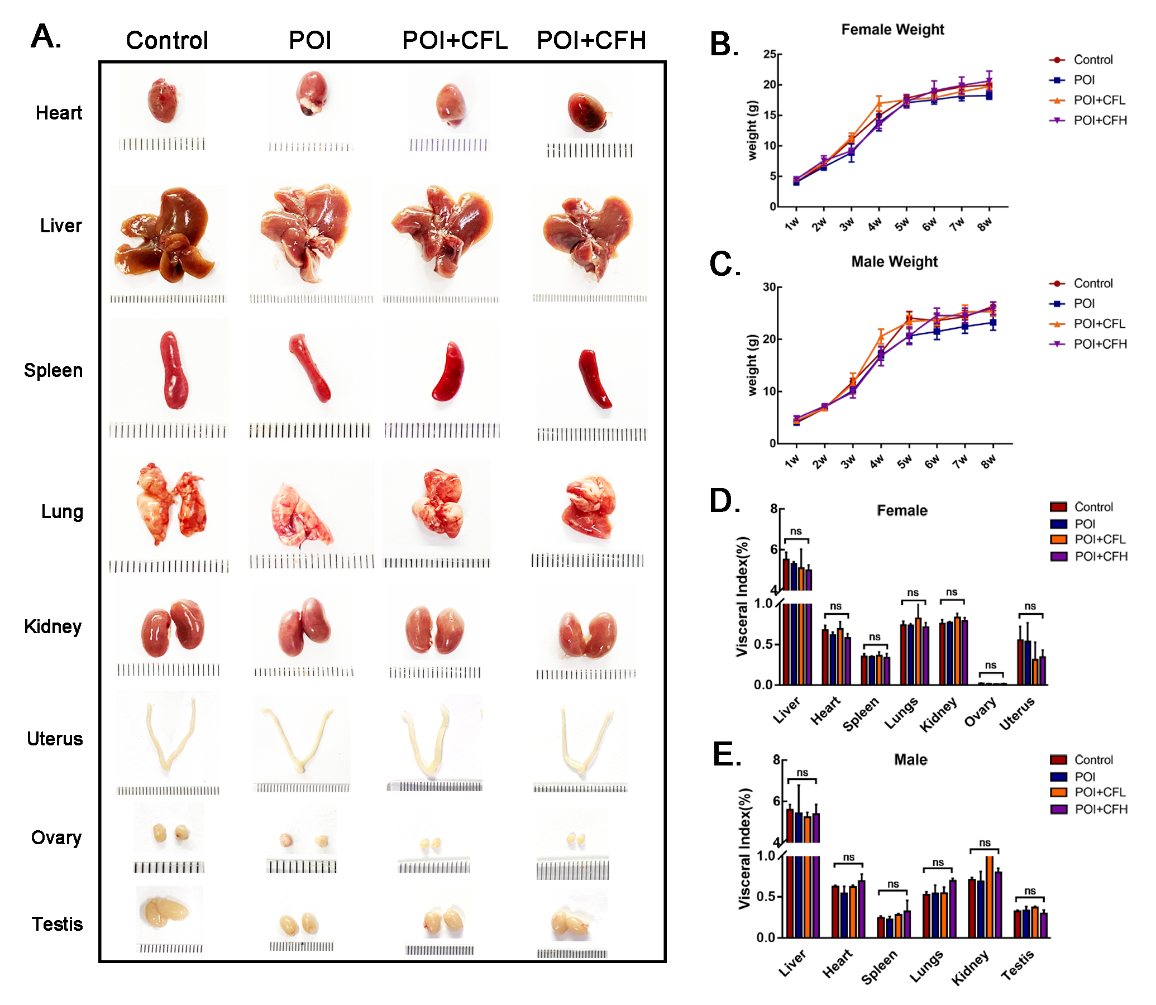
**

**Supplementary Figure S3. Biosafety assessment of CEFFE on pups.**

**(A)** The morphology of heart, live, spleen, lung, kidney, uterus, ovary and testis of female and male pups. (**B)** Dynamic monitor of the body mass of female offspring from different groups weekly (n=5). (**C)** Dynamic monitor of the body mass of male offspring from different groups weekly (n=5). **(D)** The organ index of 8 week-old female pups (n=5). **(E)** The organ index of 8 week-old male pups (n=5). Data are represented as the mean ± SD. ns: *P*>0.05, no statistical difference.


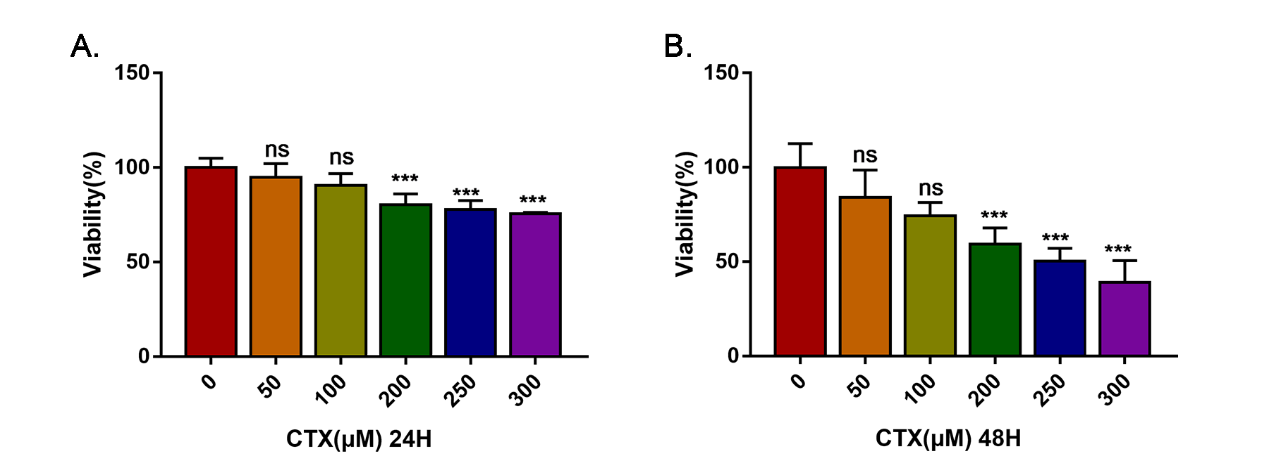


**Supplementary Figure S4. Establishment of CTX-damaged KGN cell model.**

**(A)** The viability of KGN cells treated with different concentration of CTX for 24 hours. **(B)** The viability of KGN cells treated with different concentration of CTX for 48 hours. Data are represented as the mean ± SD. ns: P >0.05, *P < 0.05, **P < 0.01,***P < 0.001.

**
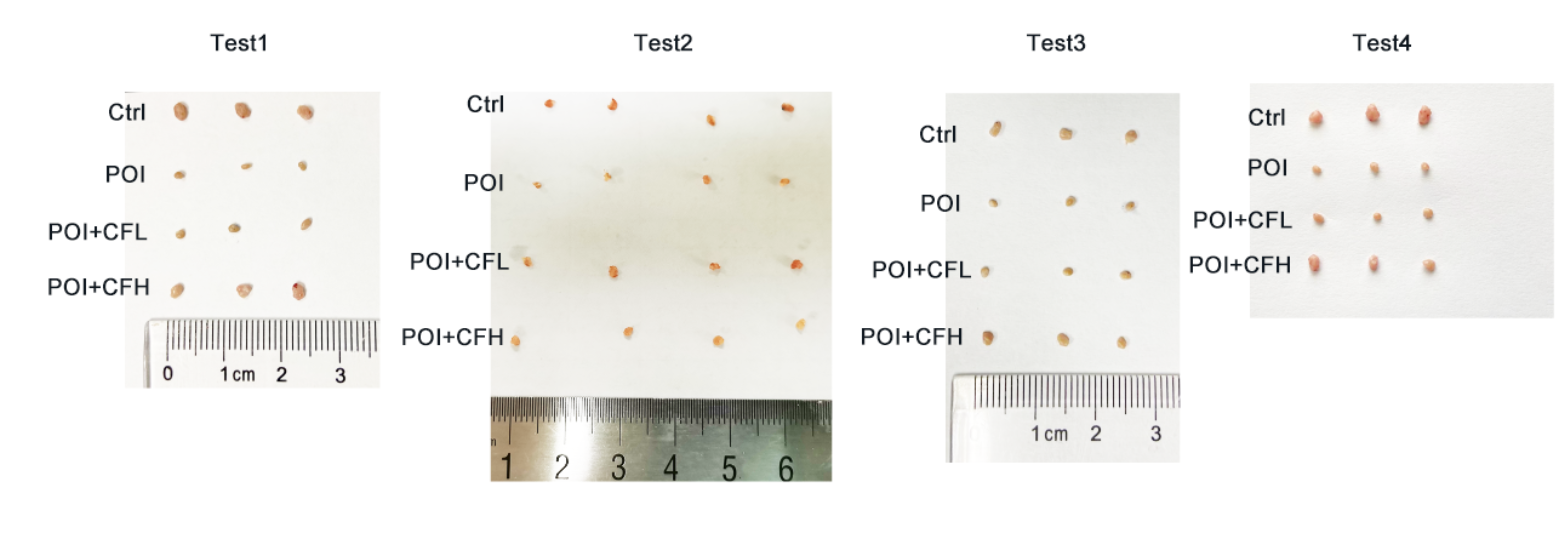
**

**Supplementary Figure S5**. All the gross morphology of ovaries from different groups with four individual experiments.


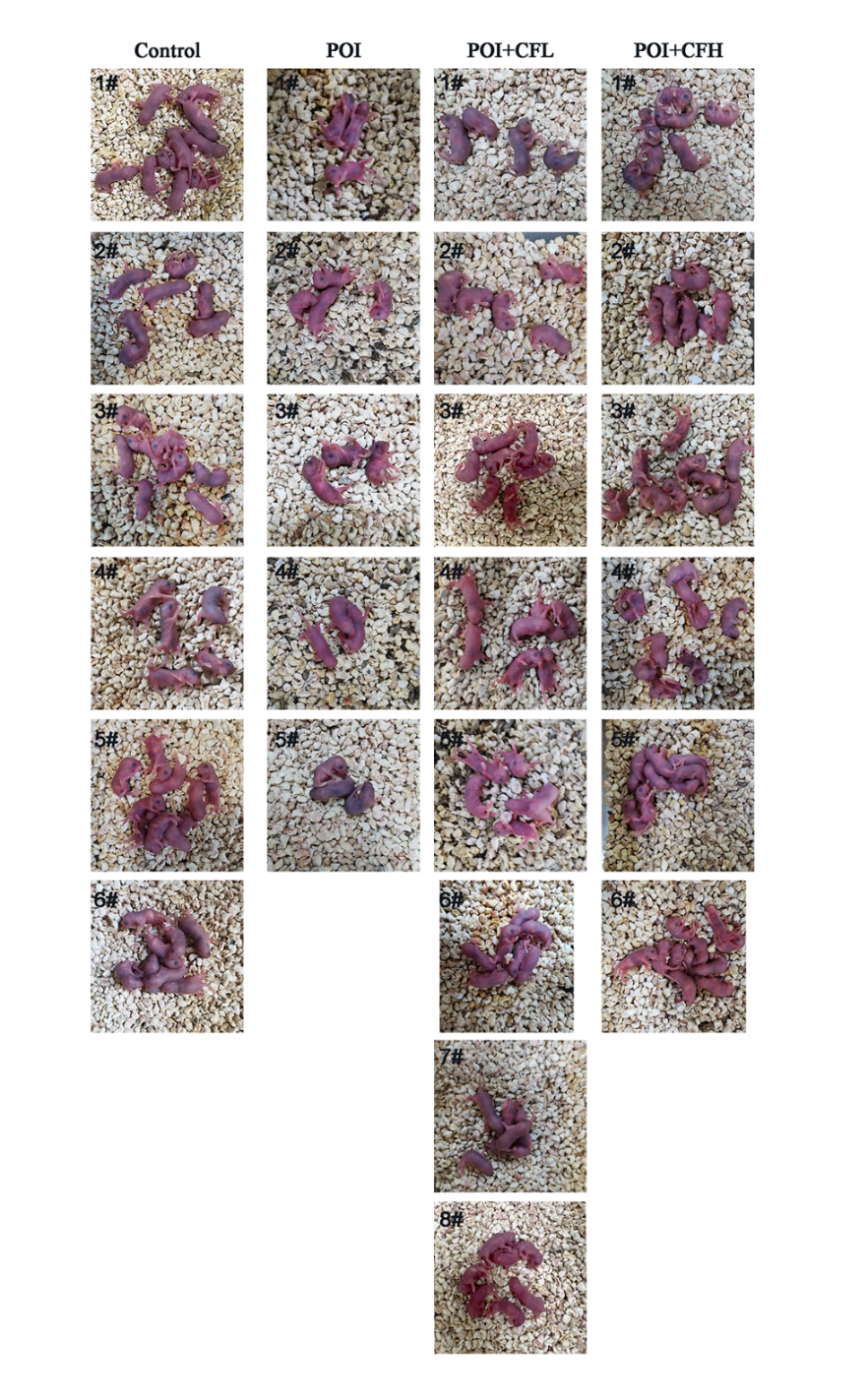


**Supplementary Figure S6.** The original images of pups from different groups.
