# Supplementary material for: Cell-free fat extract improves ovarian function and fertility in mice with premature ovarian insufficiency
Source: Stem Cell Res Ther. 2022 Jul 16;13:320. doi: 10.1186/s13287-022-03012-w (PMC9288692; doi:10.1186/s13287-022-03012-w)
Supplement: Supplementary file 3 — Additional file 3: Table S1. The detail information of antibodies used in this study. [file 13287_2022_3012_MOESM3_ESM.docx]

**Cell-free Fat Extract Improves Ovarian Function and Fertility in Mice with Premature Ovarian Insufficiency**

**Additional file 3**

**Supplementary Table. S1 The detail information of antibodies**

| **Antibody** | **Dilution ratio** | **Application** | **Product details** |
| --- | --- | --- | --- |
| Cleaved-Caspase3 | 1:400 | IHC | #9664,CST,USA |
| CD31 | 1:1000 | IHC | ab182981, Abcam, USA |
| Ki-67 | 1:200 | IHC | ab16667, Abcam, USA |
| PTEN | 1:200 | IHC | ab267787, Abcam, USA |
| P53 | 1:50 | IHC | ab131442, Abcam, USA |
| HRP-linked anti-rabbit IgG antibody | 1:100 | IHC | A0208, Beyotime, China |
| HRP-linked anti-mouse IgG antibody | 1:100 | IHC | A0216, Beyotime, China |
| GAPDH | 1:1000 | WB | #5174, CST, USA |
| Phospho-SMAD2 | 1:1000 | WB | #3108, CST, USA |
| Phospho-SMAD3 | 1:1000 | WB | #9520, CST, USA |
| SMAD2/3 | 1:1000 | WB | #8685, CST, USA |
| Bcl-2 | 1:2000 | WB | ab182858, Abcam, USA |
| Bax | 1:2000 | WB | ab182733, Abcam, USA |
| HRP-linked anti-rabbit IgG antibody | 1:3000 | WB | #7074, CST, USA |
| HRP-linked anti-mouse IgG antibody | 1:3000 | WB | #7076, CST, USA |
| FSHR | 1:50 | IF | 22665-1-AP, Proteintech, USA |
| Anti-Rabbit IgG - H&L (Alexa Fluor@488) | 1:500 | IF | #74412, CST, USA |
| Hoechst 33342 | 1:2000 | IF | C1022, Beyotime, China |
