# Supplementary material for: Cell-free fat extract improves ovarian function and fertility in mice with premature ovarian insufficiency
Source: Stem Cell Res Ther. 2022 Jul 16;13:320. doi: 10.1186/s13287-022-03012-w (PMC9288692; doi:10.1186/s13287-022-03012-w)
Supplement: Supplementary file 4 — Additional file 4: Table S2. Homologene proteins human to mouse in CEFFE. [file 13287_2022_3012_MOESM4_ESM.docx]

**Cell-free Fat Extract Improves Ovarian Function and Fertility in Mice with Premature Ovarian Insufficiency**

**Additional file 4**

**Supplementary Table S2. Homologene proteins human to mouse in CEFFE**

| **Gene name-Human** | **Gene name-Mouse** | **Gene_ID-Human** | **Gene_ID-Mouse** |
| --- | --- | --- | --- |
| SAE1 | Sae1 | 10055 | 56459 |
| ESYT1 | Esyt1 | 23344 | 23943 |
| OXCT1 | Oxct1 | 5019 | 67041 |
| TSN | Tsn | 7247 | 22099 |
| RDH5 | Rdh5 | 5959 | 19682 |
| BASP1 | Basp1 | 10409 | 70350 |
| IARS | Iars | 3376 | 105148 |
| CAND1 | Cand1 | 55832 | 71902 |
| AHCYL1 | Ahcyl1 | 10768 | 229709 |
| RPLP0 | Rplp0 | 6175 | 11837 |
| APRT | Aprt | 353 | 11821 |
| PTPN23 | Ptpn23 | 25930 | 104831 |
| MRAS | Mras | 22808 | 17532 |
| CCT8 | Cct8 | 10694 | 12469 |
| FLNC | Flnc | 2318 | 68794 |
| DARS | Dars | 1615 | 226414 |
| GNA11 | Gna11 | 2767 | 14672 |
| GOLGA3 | Golga3 | 2802 | 269682 |
| MSRB3 | Msrb3 | 253827 | 320183 |
| FASN | Fasn | 2194 | 14104 |
| EFTUD2 | Eftud2 | 9343 | 20624 |
| SERPINB9 | Serpinb9 | 5272 | 20723 |
| COL1A2 | Col1a2 | 1278 | 12843 |
| SEPT7 | Sept7 | 989 | 235072 |
| DENR | Denr | 8562 | 68184 |
| MTA2 | Mta2 | 9219 | 23942 |
| PDXK | Pdxk | 8566 | 216134 |
| PDE12 | Pde12 | 201626 | 211948 |
| LMAN1 | Lman1 | 3998 | 70361 |
| RDX | Rdx | 5962 | 19684 |
| SF3B1 | Sf3b1 | 23451 | 81898 |
| ATP5H | Atp5h | 10476 | 71679 |
| EHHADH | Ehhadh | 1962 | 74147 |
| SOD1 | Sod1 | 6647 | 20655 |
| RAB5C | Rab5c | 5878 | 19345 |
| PFDN1 | Pfdn1 | 5201 | 67199 |
| SUOX | Suox | 6821 | 211389 |
| NDUFS2 | Ndufs2 | 4720 | 226646 |
| SDHA | Sdha | 6389 | 66945 |
| GOT1 | Got1 | 2805 | 14718 |
| PSMD13 | Psmd13 | 5719 | 23997 |
| PARP1 | Parp1 | 142 | 11545 |
| CCT7 | Cct7 | 10574 | 12468 |
| COL1A1 | Col1a1 | 1277 | 12842 |
| SCAMP2 | Scamp2 | 10066 | 24044 |
| PSMD1 | Psmd1 | 5707 | 70247 |
| CS | Cs | 1431 | 12974 |
| MUT | Mut | 4594 | 17850 |
| SARS | Sars | 6301 | 20226 |
| UBA3 | Uba3 | 9039 | 22200 |
| DYNLT3 | Dynlt3 | 6990 | 67117 |
| ACAA2 | Acaa2 | 10449 | 52538 |
| HACL1 | Hacl1 | 26061 | 56794 |
| SCCPDH | Sccpdh | 51097 | 109232 |
| GAA | Gaa | 2548 | 14387 |
| AHNAK2 | Ahnak2 | 113146 | 100041194 |
| RAB7A | Rab7 | 7879 | 19349 |
| TAGLN | Tagln | 6876 | 21345 |
| MEMO1 | Memo1 | 51072 | 76890 |
| TBC1D10B | Tbc1d10b | 26000 | 68449 |
| G6PD | G6pdx | 2539 | 14381 |
| HP1BP3 | Hp1bp3 | 50809 | 15441 |
| RPL18 | Rpl18 | 6141 | 19899 |
| PSMD4 | Psmd4 | 5710 | 19185 |
| H3F3A | H3f3b | 3020 | 15081 |
| H3F3A | H3f3a | 3020 | 15078 |
| LANCL1 | Lancl1 | 10314 | 14768 |
| PCK1 | Pck1 | 5105 | 18534 |
| COPG2 | Copg2 | 26958 | 54160 |
| LRPPRC | Lrpprc | 10128 | 72416 |
| ERLIN2 | Erlin2 | 11160 | 244373 |
| AHSG | Ahsg | 197 | 11625 |
| GMPPB | Gmppb | 29925 | 331026 |
| ITGAV | Itgav | 3685 | 16410 |
| SEC13 | Sec13 | 6396 | 110379 |
| PPP5C | Ppp5c | 5536 | 19060 |
| CYBA | Cyba | 1535 | 13057 |
| SLC2A4 | Slc2a4 | 6517 | 20528 |
| STAT5B | Stat5b | 6777 | 20851 |
| NDUFA4 | Ndufa4 | 4697 | 17992 |
| RENBP | Renbp | 5973 | 19703 |
| GOT2 | Got2 | 2806 | 14719 |
| MCTS1 | Mcts1 | 28985 | 68995 |
| RPA1 | Rpa1 | 6117 | 68275 |
| EIF2S1 | Eif2s1 | 1965 | 13665 |
| DDX1 | Ddx1 | 1653 | 104721 |
| HSPG2 | Hspg2 | 3339 | 15530 |
| LAMB2 | Lamb2 | 3913 | 16779 |
| SIAE | Siae | 54414 | 22619 |
| NAE1 | Nae1 | 8883 | 234664 |
| SSB | Ssb | 6741 | 20823 |
| FOLR2 | Folr2 | 2350 | 14276 |
| S100B | S100b | 6285 | 20203 |
| ERI3 | Eri3 | 79033 | 140546 |
| CUTC | Cutc | 51076 | 66388 |
| ACTL6A | Actl6a | 86 | 56456 |
| COL12A1 | Col12a1 | 1303 | 12816 |
| ARF1 | Arf1 | 375 | 11840 |
| LHPP | Lhpp | 64077 | 76429 |
| RIPK1 | Ripk1 | 8737 | 19766 |
| IDI1 | Idi1 | 3422 | 319554 |
| UBQLN1 | Ubqln1 | 29979 | 56085 |
| PSMC4 | Psmc4 | 5704 | 23996 |
| NUDCD2 | Nudcd2 | 134492 | 52653 |
| GSS | Gss | 2937 | 14854 |
| NFU1 | Nfu1 | 27247 | 56748 |
| SKP1 | Skp1a | 6500 | 21402 |
| EGFR | Egfr | 1956 | 13649 |
| DCTN4 | Dctn4 | 51164 | 67665 |
| PRKAR2B | Prkar2b | 5577 | 19088 |
| PSMB5 | Psmb5 | 5693 | 19173 |
| H6PD | H6pd | 9563 | 100198 |
| SEC31A | Sec31a | 22872 | 69162 |
| CLTC | Cltc | 1213 | 67300 |
| C8orf82 | C030006K11Rik | 414919 | 223665 |
| XRCC6 | Xrcc6 | 2547 | 14375 |
| TPP2 | Tpp2 | 7174 | 22019 |
| PYGM | Pygm | 5837 | 19309 |
| PTPN11 | Ptpn11 | 5781 | 19247 |
| NADK2 | Nadk2 | 133686 | 68646 |
| PXDN | Pxdn | 7837 | 69675 |
| PHGDH | Phgdh | 26227 | 236539 |
| IDH1 | Idh1 | 3417 | 15926 |
| PGM1 | Pgm2 | 5236 | 72157 |
| TPI1 | Tpi1 | 7167 | 21991 |
| DDB1 | Ddb1 | 1642 | 13194 |
| PPP3CA | Ppp3ca | 5530 | 19055 |
| RBMX | Rbmx | 27316 | 19655 |
| PDHA1 | Pdha1 | 5160 | 18597 |
| CRYL1 | Cryl1 | 51084 | 68631 |
| PITRM1 | Pitrm1 | 10531 | 69617 |
| H2AFY | H2afy | 9555 | 26914 |
| TAGLN2 | Tagln2 | 8407 | 21346 |
| EIF3F | Eif3f | 8665 | 66085 |
| PHLDB1 | Phldb1 | 23187 | 102693 |
| HCFC1 | Hcfc1 | 3054 | 15161 |
| FLNB | Flnb | 2317 | 286940 |
| LYPLA1 | Lypla1 | 10434 | 18777 |
| NT5C | Nt5c | 30833 | 50773 |
| FBLN2 | Fbln2 | 2199 | 14115 |
| HSPA5 | Hspa5 | 3309 | 14828 |
| FERMT2 | Fermt2 | 10979 | 218952 |
| ACADS | Acads | 35 | 11409 |
| WDFY1 | Wdfy1 | 57590 | 69368 |
| PLIN1 | Plin1 | 5346 | 103968 |
| HSPH1 | Hsph1 | 10808 | 15505 |
| NIT1 | Nit1 | 4817 | 27045 |
| HAGH | Hagh | 3029 | 14651 |
| LDHD | Ldhd | 197257 | 52815 |
| LMOD1 | Lmod1 | 25802 | 93689 |
| COX7A1 | Cox7a1 | 1346 | 12865 |
| GBE1 | Gbe1 | 2632 | 74185 |
| ISOC2 | Isoc2a | 79763 | 664994 |
| LAMTOR5 | Lamtor5 | 10542 | 68576 |
| HSPD1 | Hspd1 | 3329 | 15510 |
| SSBP1 | Ssbp1 | 6742 | 381760 |
| TMED2 | Tmed2 | 10959 | 56334 |
| TMED2 | Gm21540 | 10959 | 100862175 |
| VCL | Vcl | 7414 | 22330 |
| ETFB | Etfb | 2109 | 110826 |
| SURF4 | Surf4 | 6836 | 20932 |
| ANXA6 | Anxa6 | 309 | 11749 |
| PSMD12 | Psmd12 | 5718 | 66997 |
| ERH | Erh | 2079 | 13877 |
| UQCRFS1 | Uqcrfs1 | 7386 | 66694 |
| UCHL3 | Uchl3 | 7347 | 50933 |
| LCP1 | Lcp1 | 3936 | 18826 |
| VCP | Vcp | 7415 | 269523 |
| PSMB10 | Psmb10 | 5699 | 19171 |
| CPNE1 | Cpne1 | 8904 | 266692 |
| UGGT1 | Uggt1 | 56886 | 320011 |
| S100A11 | Gm5068 | 6282 | 277089 |
| FARSA | Farsa | 2193 | 66590 |
| RBP4 | Rbp4 | 5950 | 19662 |
| LMAN2 | Lman2 | 10960 | 66890 |
| LYPLAL1 | Lyplal1 | 127018 | 226791 |
| APIP | Apip | 51074 | 56369 |
| PPP1CA | Ppp1ca | 5499 | 19045 |
| SETD7 | Setd7 | 80854 | 73251 |
| PA2G4 | Pa2g4 | 5036 | 18813 |
| NTM | Ntm | 50863 | 235106 |
| PDCD6IP | Pdcd6ip | 10015 | 18571 |
| OPA1 | Opa1 | 4976 | 74143 |
| PTGR1 | Ptgr1 | 22949 | 67103 |
| BIN1 | Bin1 | 274 | 30948 |
| PEX6 | Pex6 | 5190 | 224824 |
| ACY1 | Acy1 | 95 | 109652 |
| SORBS1 | Sorbs1 | 10580 | 20411 |
| KPNB1 | Kpnb1 | 3837 | 16211 |
| TPD52L2 | Tpd52l2 | 7165 | 66314 |
| GSTM4 | Gstm4 | 2948 | 14865 |
| HSPB1 | Hspb1 | 3315 | 15507 |
| AKAP2 | Akap2 | 11217 | 11641 |
| ASPSCR1 | Aspscr1 | 79058 | 68938 |
| HNRNPH2 | Hnrnph2 | 3188 | 56258 |
| RABEP1 | Rabep1 | 9135 | 54189 |
| AMBP | Ambp | 259 | 11699 |
| ECHDC3 | Echdc3 | 79746 | 67856 |
| RPL4 | Rpl4 | 6124 | 67891 |
| KARS | Kars | 3735 | 85305 |
| CFD | Cfd | 1675 | 11537 |
| FAM213A | Fam213a | 84293 | 70564 |
| GALNS | Galns | 2588 | 50917 |
| PSMA2 | Psma2 | 5683 | 19166 |
| FKBP9 | Fkbp9 | 11328 | 27055 |
| KANK4 | Kank4 | 163782 | 242553 |
| IDH3B | Idh3b | 3420 | 170718 |
| EIF5A | Eif5a | 1984 | 276770 |
| CACNA2D1 | Cacna2d1 | 781 | 12293 |
| COL5A3 | Col5a3 | 50509 | 53867 |
| NME2 | Nme2 | 4831 | 18103 |
| GNAI1 | Gnai1 | 2770 | 14677 |
| ACTN2 | Actn2 | 88 | 11472 |
| DLG1 | Dlg1 | 1739 | 13383 |
| CA2 | Car2 | 760 | 12349 |
| UBXN1 | Ubxn1 | 51035 | 225896 |
| PHYKPL | Phykpl | 85007 | 72947 |
| NDUFB11 | Ndufb11 | 54539 | 104130 |
| STMN1 | Stmn1 | 3925 | 16765 |
| DUSP23 | Dusp23 | 54935 | 68440 |
| PXN | Pxn | 5829 | 19303 |
| PSMB9 | Psmb9 | 5698 | 16912 |
| EIF4B | Eif4b | 1975 | 75705 |
| TPP1 | Tpp1 | 1200 | 12751 |
| MRPS36 | Mrps36 | 92259 | 66128 |
| RPA3 | Rpa3 | 6119 | 68240 |
| SERPINB6 | Serpinb6a | 5269 | 20719 |
| EEF1G | Eef1g | 1937 | 67160 |
| TSTD1 | Tstd1 | 100131187 | 226654 |
| CORO1C | Coro1c | 23603 | 23790 |
| GC | Gc | 2638 | 14473 |
| RMDN1 | Rmdn1 | 51115 | 66302 |
| COX5A | Cox5a | 9377 | 12858 |
| RRAS | Rras | 6237 | 20130 |
| MANF | Manf | 7873 | 74840 |
| DHX15 | Dhx15 | 1665 | 13204 |
| AARS | Aars | 16 | 234734 |
| PHPT1 | Phpt1 | 29085 | 75454 |
| PRDX2 | Prdx2 | 7001 | 21672 |
| HSBP1 | Hsbp1 | 3281 | 68196 |
| CPT2 | Cpt2 | 1376 | 12896 |
| OPLAH | Oplah | 26873 | 75475 |
| SELENBP1 | Selenbp1 | 8991 | 20341 |
| SELENBP1 | Selenbp2 | 8991 | 20342 |
| TPK1 | Tpk1 | 27010 | 29807 |
| HYOU1 | Hyou1 | 10525 | 12282 |
| EIF3A | Eif3a | 8661 | 13669 |
| HSPB6 | Hspb6 | 126393 | 243912 |
| CD59 | Cd59b | 966 | 333883 |
| ARSA | Arsa | 410 | 11883 |
| UBE2I | Ube2i | 7329 | 22196 |
| LIMS1 | Lims1 | 3987 | 110829 |
| WDR44 | Wdr44 | 54521 | 72404 |
| GART | Gart | 2618 | 14450 |
| AHNAK | Ahnak | 79026 | 66395 |
| ACTG1 | Actg1 | 71 | 11465 |
| MCCC2 | Mccc2 | 64087 | 78038 |
| CTSD | Ctsd | 1509 | 13033 |
| CAPN2 | Capn2 | 824 | 12334 |
| CKAP4 | Ckap4 | 10970 | 216197 |
| PAFAH1B3 | Pafah1b3 | 5050 | 18476 |
| NCKAP1 | Nckap1 | 10787 | 50884 |
| HOOK3 | Hook3 | 84376 | 320191 |
| GLB1 | Glb1 | 2720 | 12091 |
| NUP214 | Nup214 | 8021 | 227720 |
| SMARCC2 | Smarcc2 | 6601 | 68094 |
| PAK2 | Pak2 | 5062 | 224105 |
| EIF4A2 | Eif4a2 | 1974 | 13682 |
| CSNK2B | Csnk2b | 1460 | 13001 |
| CAPRIN1 | Caprin1 | 4076 | 53872 |
| DECR1 | Decr1 | 1666 | 67460 |
| VAT1 | Vat1 | 10493 | 26949 |
| PPP6C | Ppp6c | 5537 | 67857 |
| FKBP4 | Fkbp4 | 2288 | 14228 |
| JMJD7 | Jmjd7 | 100137047 | 433466 |
| TCOF1 | Tcof1 | 6949 | 21453 |
| CORO1A | Coro1a | 11151 | 12721 |
| DPYSL2 | Dpysl2 | 1808 | 12934 |
| RAB10 | Rab10 | 10890 | 19325 |
| CLIC1 | Clic1 | 1192 | 114584 |
| KANK2 | Kank2 | 25959 | 235041 |
| RPL5 | Rpl5 | 6125 | 100503670 |
| PSMD14 | Psmd14 | 10213 | 59029 |
| OSBPL1A | Osbpl1a | 114876 | 64291 |
| TTC1 | Ttc1 | 7265 | 66827 |
| UQCR10 | Uqcr10 | 29796 | 66152 |
| KHSRP | Khsrp | 8570 | 16549 |
| NOMO1 | Nomo1 | 23420 | 211548 |
| CD2AP | Cd2ap | 23607 | 12488 |
| CTBP1 | Ctbp1 | 1487 | 13016 |
| HNRNPC | Hnrnpc | 3183 | 15381 |
| CPNE3 | Cpne3 | 8895 | 70568 |
| HSPA12A | Hspa12a | 259217 | 73442 |
| ASPA | Aspa | 443 | 11484 |
| STAT3 | Stat3 | 6774 | 20848 |
| GRN | Grn | 2896 | 14824 |
| GPD1L | Gpd1l | 23171 | 333433 |
| FUCA1 | Fuca1 | 2517 | 71665 |
| GLG1 | Glg1 | 2734 | 20340 |
| NRP1 | Nrp1 | 8829 | 18186 |
| MOB4 | Mob4 | 25843 | 19070 |
| UGDH | Ugdh | 7358 | 22235 |
| HSPA1B | Hspa1a | 3304 | 193740 |
| EIF5B | Eif5b | 9669 | 226982 |
| CD248 | Cd248 | 57124 | 70445 |
| CKB | Ckb | 1152 | 12709 |
| ACADM | Acadm | 34 | 11364 |
| MOGS | Mogs | 7841 | 57377 |
| PSMA6 | Psma6 | 5687 | 26443 |
| FABP4 | Fabp4 | 2167 | 11770 |
| WARS | Wars | 7453 | 22375 |
| YARS | Yars | 8565 | 107271 |
| REXO2 | Rexo2 | 25996 | 104444 |
| DCN | Dcn | 1634 | 13179 |
| MECR | Mecr | 51102 | 26922 |
| FAM98A | Fam98a | 25940 | 72722 |
| GSTM3 | Gstm5 | 2947 | 14866 |
| NPM1 | Npm1 | 4869 | 18148 |
| UBE2N | Ube2n | 7334 | 93765 |
| RAD23A | Rad23a | 5886 | 19358 |
| DBN1 | Dbn1 | 1627 | 56320 |
| FAM129B | Fam129b | 64855 | 227737 |
| NANS | Nans | 54187 | 94181 |
| NPLOC4 | Nploc4 | 55666 | 217365 |
| ABHD10 | Abhd10 | 55347 | 213012 |
| SRI | Sri | 6717 | 109552 |
| PNPO | Pnpo | 55163 | 103711 |
| CAV2 | Cav2 | 858 | 12390 |
| MYCBP | Mycbp | 26292 | 56309 |
| GRB2 | Grb2 | 2885 | 14784 |
| MAT2B | Mat2b | 27430 | 108645 |
| CD44 | Cd44 | 960 | 12505 |
| LAP3 | Lap3 | 51056 | 66988 |
| TK2 | Tk2 | 7084 | 57813 |
| AGL | Agl | 178 | 77559 |
| LRPAP1 | Lrpap1 | 4043 | 16976 |
| PPP1CB | Ppp1cb | 5500 | 19046 |
| VPS25 | Vps25 | 84313 | 28084 |
| TRIP6 | Trip6 | 7205 | 22051 |
| SHPK | Shpk | 23729 | 74637 |
| NHLRC3 | Nhlrc3 | 387921 | 212114 |
| KDELC2 | Kdelc2 | 143888 | 68304 |
| ATP6V1A | Atp6v1a | 523 | 11964 |
| PECR | Pecr | 55825 | 111175 |
| SLC9A3R2 | Slc9a3r2 | 9351 | 65962 |
| TUBA1B | Tuba1b | 10376 | 22143 |
| ENO1 | Gm5506 | 2023 | 433182 |
| ENO1 | Eno1 | 2023 | 13806 |
| CREG1 | Creg1 | 8804 | 433375 |
| KATNAL2 | Katnal2 | 83473 | 71206 |
| PCBP2 | Pcbp2 | 5094 | 18521 |
| DPT | Dpt | 1805 | 56429 |
| LTA4H | Lta4h | 4048 | 16993 |
| ESYT2 | Esyt2 | 57488 | 52635 |
| NUCB2 | Nucb2 | 4925 | 53322 |
| DDHD2 | Ddhd2 | 23259 | 72108 |
| HDLBP | Hdlbp | 3069 | 110611 |
| CLU | Clu | 1191 | 12759 |
| PPIB | Ppib | 5479 | 19035 |
| FBLN5 | Fbln5 | 10516 | 23876 |
| CNN1 | Cnn1 | 1264 | 12797 |
| HAAO | Haao | 23498 | 107766 |
| GIMAP4 | Gimap4 | 55303 | 107526 |
| ABHD14B | Abhd14b | 84836 | 76491 |
| EEF1D | Eef1d | 1936 | 66656 |
| ARHGAP1 | Arhgap1 | 392 | 228359 |
| ALAD | Alad | 210 | 17025 |
| PCCB | Pccb | 5096 | 66904 |
| COMMD9 | Commd9 | 29099 | 76501 |
| PMPCB | Pmpcb | 9512 | 73078 |
| PSMD3 | Psmd3 | 5709 | 22123 |
| DAG1 | Dag1 | 1605 | 13138 |
| LAMC1 | Lamc1 | 3915 | 226519 |
| UBA6 | Uba6 | 55236 | 231380 |
| TST | Tst | 7263 | 22117 |
| ITIH4 | Itih4 | 3700 | 16427 |
| CHCHD3 | Chchd3 | 54927 | 66075 |
| PRKACB | Prkacb | 5567 | 18749 |
| ITGB1 | Itgb1 | 3688 | 16412 |
| ARPC2 | Arpc2 | 10109 | 76709 |
| VPS26B | Vps26b | 112936 | 69091 |
| AP1S1 | Ap1s1 | 1174 | 11769 |
| MRC1 | Mrc1 | 4360 | 17533 |
| SF3B3 | Sf3b3 | 23450 | 101943 |
| PITPNB | Pitpnb | 23760 | 56305 |
| SMS | Sms | 6611 | 20603 |
| SMS | Gm14680 | 6611 | 671878 |
| BSG | Bsg | 682 | 12215 |
| ADK | Adk | 132 | 11534 |
| DTD1 | Dtd1 | 92675 | 66044 |
| HSD17B10 | Hsd17b10 | 3028 | 15108 |
| PSMB2 | Psmb2 | 5690 | 26445 |
| PRKAR2A | Prkar2a | 5576 | 19087 |
| PPP1R14A | Ppp1r14a | 94274 | 68458 |
| LMNB2 | Lmnb2 | 84823 | 16907 |
| SGCD | Sgcd | 6444 | 24052 |
| COQ9 | Coq9 | 57017 | 67914 |
| TYMP | Tymp | 1890 | 72962 |
| HYPK | Hypk | 25764 | 67693 |
| DDR2 | Ddr2 | 4921 | 18214 |
| PSMD11 | Psmd11 | 5717 | 69077 |
| CNP | Cnp | 1267 | 12799 |
| C20orf27 | 1700037H04Rik | 54976 | 67326 |
| NONO | Nono | 4841 | 53610 |
| DDT | Ddt | 1652 | 13202 |
| SNX5 | Snx5 | 27131 | 69178 |
| GYG1 | Gyg | 2992 | 27357 |
| NPC2 | Npc2 | 10577 | 67963 |
| CD163 | Cd163 | 9332 | 93671 |
| IGF2R | Igf2r | 3482 | 16004 |
| SUMF2 | Sumf2 | 25870 | 67902 |
| DYNLL1 | Dynll1 | 8655 | 56455 |
| ALDH4A1 | Aldh4a1 | 8659 | 212647 |
| ASNA1 | Asna1 | 439 | 56495 |
| CCT3 | Cct3 | 7203 | 12462 |
| HNMT | Hnmt | 3176 | 140483 |
| SORBS3 | Sorbs3 | 10174 | 20410 |
| GRHPR | Grhpr | 9380 | 76238 |
| SRM | Srm | 6723 | 20810 |
| CAMK2D | Camk2d | 817 | 108058 |
| SRSF7 | Srsf7 | 6432 | 225027 |
| ENAH | Enah | 55740 | 13800 |
| MSN | Msn | 4478 | 17698 |
| CPM | Cpm | 1368 | 70574 |
| VPS35 | Vps35 | 55737 | 65114 |
| THEM6 | Them6 | 51337 | 223626 |
| CACNG6 | Cacng6 | 59285 | 54378 |
| USP15 | Usp15 | 9958 | 14479 |
| THOP1 | Thop1 | 7064 | 50492 |
| CYC1 | Cyc1 | 1537 | 66445 |
| G3BP1 | G3bp1 | 10146 | 27041 |
| CHCHD4 | Chchd4 | 131474 | 72170 |
| ARPC3 | Arpc3 | 10094 | 56378 |
| KHDRBS1 | Khdrbs1 | 10657 | 20218 |
| SLC25A3 | Slc25a3 | 5250 | 18674 |
| AMDHD2 | Amdhd2 | 51005 | 245847 |
| TXN | Txn1 | 7295 | 22166 |
| DIABLO | Diablo | 56616 | 66593 |
| COPA | Copa | 1314 | 12847 |
| GLRX3 | Glrx3 | 10539 | 30926 |
| PSMD10 | Psmd10 | 5716 | 53380 |
| HMGCL | Hmgcl | 3155 | 15356 |
| OLA1 | Ola1 | 29789 | 67059 |
| TRIOBP | Triobp | 11078 | 110253 |
| DNPEP | Dnpep | 23549 | 13437 |
| ST13 | St13 | 6767 | 70356 |
| SEC23A | Sec23a | 10484 | 20334 |
| NUDC | Nudc | 10726 | 18221 |
| PDLIM7 | Pdlim7 | 9260 | 67399 |
| VTA1 | Vta1 | 51534 | 66201 |
| HSPE1 | Hspe1 | 3336 | 15528 |
| CAPG | Capg | 822 | 12332 |
| PSMA5 | Psma5 | 5686 | 26442 |
| GNG12 | Gng12 | 55970 | 14701 |
| PSMD5 | Psmd5 | 5711 | 66998 |
| BLVRA | Blvra | 644 | 109778 |
| HSPA8 | Hspa8 | 3312 | 15481 |
| COPZ1 | Copz1 | 22818 | 56447 |
| GNAI3 | Gnai3 | 2773 | 14679 |
| DERA | Dera | 51071 | 232449 |
| TMEM205 | Tmem205 | 374882 | 235043 |
| AGPAT2 | Agpat2 | 10555 | 67512 |
| PYGB | Pygb | 5834 | 110078 |
| CAPZA2 | Capza2 | 830 | 12343 |
| CTNNBIP1 | Ctnnbip1 | 56998 | 67087 |
| HNRNPUL1 | Hnrnpul1 | 11100 | 232989 |
| ABLIM2 | Ablim2 | 84448 | 231148 |
| FAM120A | Fam120a | 23196 | 218236 |
| ACTN4 | Actn4 | 81 | 60595 |
| CPA3 | Cpa3 | 1359 | 12873 |
| LPP | Lpp | 4026 | 210126 |
| AKAP12 | Akap12 | 9590 | 83397 |
| MAPK1 | Mapk1 | 5594 | 26413 |
| S100A6 | S100a6 | 6277 | 20200 |
| COPS7A | Cops7a | 50813 | 26894 |
| GCLC | Gclc | 2729 | 14629 |
| GNAI2 | Gnai2 | 2771 | 14678 |
| PPL | Ppl | 5493 | 19041 |
| UBA1 | Uba1 | 7317 | 22201 |
| SCRN2 | Scrn2 | 90507 | 217140 |
| EIF3J | Eif3j1 | 8669 | 78655 |
| EIF3J | Eif3j2 | 8669 | 100042807 |
| SPATA7 | Spata7 | 55812 | 104871 |
| TTR | Ttr | 7276 | 22139 |
| LAMA4 | Lama4 | 3910 | 16775 |
| ATP2B4 | Atp2b4 | 493 | 381290 |
| HDGF | Hdgf | 3068 | 15191 |
| CNPY2 | Cnpy2 | 10330 | 56530 |
| RAP1GDS1 | Rap1gds1 | 5910 | 229877 |
| EHD1 | Ehd1 | 10938 | 13660 |
| TOMM22 | Tomm22 | 56993 | 223696 |
| LMNB1 | Lmnb1 | 4001 | 16906 |
| PPP2R1A | Ppp2r1a | 5518 | 51792 |
| GATM | Gatm | 2628 | 67092 |
| DPH5 | Dph5 | 51611 | 69740 |
| CUL3 | Cul3 | 8452 | 26554 |
| GLRX | Glrx | 2745 | 93692 |
| TTLL12 | Ttll12 | 23170 | 223723 |
| PSMD9 | Psmd9 | 5715 | 67151 |
| SNRPF | Snrpf | 6636 | 69878 |
| PROCR | Procr | 10544 | 19124 |
| DCLK1 | Dclk1 | 9201 | 13175 |
| ALDH2 | Aldh2 | 217 | 11669 |
| COX4I1 | Cox4i1 | 1327 | 12857 |
| ACOT1 | Acot3 | 641371 | 171281 |
| TUBA1A | Tuba1a | 7846 | 22142 |
| RAC1 | Rac1 | 5879 | 19353 |
| IPO5 | Ipo5 | 3843 | 70572 |
| RAN | Ran | 5901 | 19384 |
| CTSC | Ctsc | 1075 | 13032 |
| ARF4 | Arf4 | 378 | 11843 |
| ITGA7 | Itga7 | 3679 | 16404 |
| BPHL | Bphl | 670 | 68021 |
| VDAC1 | Vdac1 | 7416 | 22333 |
| ORMDL3 | Ormdl3 | 94103 | 66612 |
| EEA1 | Eea1 | 8411 | 216238 |
| SERPINH1 | Serpinh1 | 871 | 12406 |
| PRMT5 | Prmt5 | 10419 | 27374 |
| SEPT8 | Sept8 | 23176 | 20362 |
| SCRN1 | Scrn1 | 9805 | 69938 |
| NME3 | Nme3 | 4832 | 79059 |
| GNB2 | Gnb2 | 2783 | 14693 |
| SERPINB1 | Serpinb1a | 1992 | 66222 |
| PALMD | Palmd | 54873 | 114301 |
| ADH1C | Adh1 | 126 | 11522 |
| GSTM2 | Gstm7 | 2946 | 68312 |
| CNBP | Cnbp | 7555 | 12785 |
| CPQ | Cpq | 10404 | 54381 |
| TUBB6 | Tubb6 | 84617 | 67951 |
| REEP6 | Reep6 | 92840 | 70335 |
| PSMC6 | Psmc6 | 5706 | 67089 |
| EML1 | Eml1 | 2009 | 68519 |
| ITGA6 | Itga6 | 3655 | 16403 |
| PBLD | Pbld1 | 64081 | 68371 |
| AP2S1 | Ap2s1 | 1175 | 232910 |
| RBBP7 | Rbbp7 | 5931 | 245688 |
| FNTA | Fnta | 2339 | 14272 |
| NSF | Nsf | 4905 | 18195 |
| FTH1 | Fth1 | 2495 | 14319 |
| HTRA2 | Htra2 | 27429 | 64704 |
| PSMA1 | Psma1 | 5682 | 26440 |
| KLHL31 | Klhl31 | 401265 | 244923 |
| NOL3 | Nol3 | 8996 | 78688 |
| PGM5 | Pgm5 | 5239 | 226041 |
| PARVA | Parva | 55742 | 57342 |
| GANAB | Ganab | 23193 | 14376 |
| VCAN | Vcan | 1462 | 13003 |
| DNAJB2 | Dnajb2 | 3300 | 56812 |
| SOD3 | Sod3 | 6649 | 20657 |
| COL6A3 | Col6a3 | 1293 | 12835 |
| TUBB | Tubb5 | 203068 | 22154 |
| CIAPIN1 | Ciapin1 | 57019 | 109006 |
| DDAH1 | Ddah1 | 23576 | 69219 |
| SH3BGRL3 | Sh3bgrl3 | 83442 | 73723 |
| AP3D1 | Ap3d1 | 8943 | 11776 |
| VASP | Vasp | 7408 | 22323 |
| CARS | Cars | 833 | 27267 |
| SH3BGRL | Sh3bgrl | 6451 | 56726 |
| MAVS | Mavs | 57506 | 228607 |
| PRDX5 | Prdx5 | 25824 | 54683 |
| HARS | Hars | 3035 | 15115 |
| COMMD8 | Commd8 | 54951 | 27784 |
| ALDOA | Aldoa | 226 | 11674 |
| APCS | Apcs | 325 | 20219 |
| RPRD1B | Rprd1b | 58490 | 70470 |
| BROX | Brox | 148362 | 71678 |
| GYS1 | Gys1 | 2997 | 14936 |
| GSPT1 | Gspt1 | 2935 | 14852 |
| HEPACAM | Hepacam | 220296 | 72927 |
| C1orf123 | 0610037L13Rik | 54987 | 74098 |
| ALDH6A1 | Aldh6a1 | 4329 | 104776 |
| AK1 | Ak1 | 203 | 11636 |
| CD5L | Cd5l | 922 | 11801 |
| PSMC1 | Psmc1 | 5700 | 19179 |
| USP5 | Usp5 | 8078 | 22225 |
| NLN | Nln | 57486 | 75805 |
| HCLS1 | Hcls1 | 3059 | 15163 |
| PFAS | Pfas | 5198 | 237823 |
| SNRPD3 | Snrpd3 | 6634 | 67332 |
| NT5DC1 | Nt5dc1 | 221294 | 319638 |
| PRPF19 | Prpf19 | 27339 | 28000 |
| GORASP2 | Gorasp2 | 26003 | 70231 |
| LIMA1 | Lima1 | 51474 | 65970 |
| SEPT2 | Sept2 | 4735 | 18000 |
| CTSB | Ctsb | 1508 | 13030 |
| IFI35 | Ifi35 | 3430 | 70110 |
| ARHGDIA | Arhgdia | 396 | 192662 |
| TECR | Tecr | 9524 | 106529 |
| ATP6V1B2 | Atp6v1b2 | 526 | 11966 |
| NDUFS1 | Ndufs1 | 4719 | 227197 |
| CLIC4 | Clic4 | 25932 | 29876 |
| PSMC2 | Psmc2 | 5701 | 19181 |
| P4HB | P4hb | 5034 | 18453 |
| RAB14 | Rab14 | 51552 | 68365 |
| IDH3A | Idh3a | 3419 | 67834 |
| SAMHD1 | Samhd1 | 25939 | 56045 |
| COMMD3 | Commd3 | 23412 | 12238 |
| STX12 | Stx12 | 23673 | 100226 |
| VPS29 | Vps29 | 51699 | 56433 |
| CAV1 | Cav1 | 857 | 12389 |
| SERPINA1 | Serpina1e | 5265 | 20704 |
| SERPINA1 | Serpina1a | 5265 | 20700 |
| SERPINA1 | Serpina1c | 5265 | 20702 |
| SERPINA1 | Serpina1b | 5265 | 20701 |
| SERPINA1 | Serpina1d | 5265 | 20703 |
| HBG2 | Hbb-y | 3048 | 15135 |
| CYB5B | Cyb5b | 80777 | 66427 |
| LDHB | Ldhb | 3945 | 16832 |
| ACTN1 | Actn1 | 87 | 109711 |
| COL14A1 | Col14a1 | 7373 | 12818 |
| SCP2 | Scp2 | 6342 | 20280 |
| ACTB | Actb | 60 | 11461 |
| TMEM43 | Tmem43 | 79188 | 74122 |
| DLD | Dld | 1738 | 13382 |
| ATP5O | Atp5o | 539 | 28080 |
| HNRNPUL2 | Hnrnpul2 | 221092 | 68693 |
| C11orf54 | 4931406C07Rik | 28970 | 70984 |
| DYNC1H1 | Dync1h1 | 1778 | 13424 |
| DNAJB4 | Dnajb4 | 11080 | 67035 |
| GUK1 | Guk1 | 2987 | 14923 |
| ETFDH | Etfdh | 2110 | 66841 |
| CTSS | Ctss | 1520 | 13040 |
| DLST | Dlst | 1743 | 78920 |
| LZIC | Lzic | 84328 | 69151 |
| BCAP31 | Bcap31 | 10134 | 27061 |
| EPRS | Eprs | 2058 | 107508 |
| AOC3 | Aoc3 | 8639 | 11754 |
| PBXIP1 | Pbxip1 | 57326 | 229534 |
| CNN3 | Cnn3 | 1266 | 71994 |
| DPYSL3 | Dpysl3 | 1809 | 22240 |
| DCTN3 | Dctn3 | 11258 | 53598 |
| SGTA | Sgta | 6449 | 52551 |
| S100A13 | S100a13 | 6284 | 20196 |
| FAM49B | Fam49b | 51571 | 223601 |
| CACYBP | Cacybp | 27101 | 12301 |
| MYH10 | Myh10 | 4628 | 77579 |
| PLXNB2 | Plxnb2 | 23654 | 140570 |
| ACAT1 | Acat1 | 38 | 110446 |
| TTN | Ttn | 7273 | 22138 |
| TXNDC17 | Txndc17 | 84817 | 52700 |
| TUBA1C | Tuba1c | 84790 | 22146 |
| PDLIM5 | Pdlim5 | 10611 | 56376 |
| PYCARD | Pycard | 29108 | 66824 |
| PPIH | Ppih | 10465 | 66101 |
| RARS | Rars | 5917 | 104458 |
| TPM3 | Tpm3 | 7170 | 59069 |
| AK2 | Ak2 | 204 | 11637 |
| SNX6 | Snx6 | 58533 | 72183 |
| COBLL1 | Cobll1 | 22837 | 319876 |
| HSD17B4 | Hsd17b4 | 3295 | 15488 |
| PGLS | Pgls | 25796 | 66171 |
| DMD | Dmd | 1756 | 13405 |
| EPS15 | Eps15 | 2060 | 13858 |
| DYNLRB1 | Dynlrb1 | 83658 | 67068 |
| SORD | Sord | 6652 | 20322 |
| QPRT | Qprt | 23475 | 67375 |
| UBLCP1 | Ublcp1 | 134510 | 79560 |
| HSP90AB1 | Hsp90ab1 | 3326 | 15516 |
| DDI2 | Ddi2 | 84301 | 68817 |
| NDRG2 | Ndrg2 | 57447 | 29811 |
| PLS3 | Pls3 | 5358 | 102866 |
| LNPEP | Lnpep | 4012 | 240028 |
| CORO1B | Coro1b | 57175 | 23789 |
| SPECC1 | Specc1 | 92521 | 432572 |
| UQCRC1 | Uqcrc1 | 7384 | 22273 |
| ANKRD40 | Ankrd40 | 91369 | 71452 |
| PSME1 | Psme1 | 5720 | 19186 |
| ATIC | Atic | 471 | 108147 |
| PRDX6 | Prdx6 | 9588 | 11758 |
| TBCA | Tbca | 6902 | 21371 |
| YWHAZ | Ywhaz | 7534 | 22631 |
| CTTN | Cttn | 2017 | 13043 |
| ACO2 | Aco2 | 50 | 11429 |
| NCL | Ncl | 4691 | 17975 |
| PSMB1 | Psmb1 | 5689 | 19170 |
| IDH2 | Idh2 | 3418 | 269951 |
| RTN3 | Rtn3 | 10313 | 20168 |
| FGA | Fga | 2243 | 14161 |
| S100A1 | S100a1 | 6271 | 20193 |
| ACTR3 | Actr3 | 10096 | 74117 |
| ITGA1 | Itga1 | 3672 | 109700 |
| RELA | Rela | 5970 | 19697 |
| VPS26A | Vps26a | 9559 | 30930 |
| SLIRP | Slirp | 81892 | 380773 |
| TALDO1 | Taldo1 | 6888 | 21351 |
| DCPS | Dcps | 28960 | 69305 |
| TCP1 | Tcp1 | 6950 | 21454 |
| PSMC5 | Psmc5 | 5705 | 19184 |
| EIF3H | Eif3h | 8667 | 68135 |
| SEPT9 | Sept9 | 10801 | 53860 |
| IFI30 | Ifi30 | 10437 | 65972 |
| DPYD | Dpyd | 1806 | 99586 |
| EHD4 | Ehd4 | 30844 | 98878 |
| RAB11B | Rab11b | 9230 | 19326 |
| SAA1 | Saa1 | 6288 | 20208 |
| SAA1 | Saa2 | 6288 | 20209 |
| SUMO1 | Sumo1 | 7341 | 22218 |
| PPIA | Ppia | 5478 | 268373 |
| AKR1B1 | Akr1b3 | 231 | 11677 |
| TRIM28 | Trim28 | 10155 | 21849 |
| HNRNPK | Hnrnpk | 3190 | 15387 |
| SUMO2 | Sumo2 | 6613 | 170930 |
| TJP1 | Tjp1 | 7082 | 21872 |
| PGM3 | Pgm3 | 5238 | 109785 |
| AKR1C2 | Akr1c21 | 1646 | 77337 |
| ECH1 | Ech1 | 1891 | 51798 |
| ACSS2 | Acss2 | 55902 | 60525 |
| CTSZ | Ctsz | 1522 | 64138 |
| CYGB | Cygb | 114757 | 114886 |
| LGALS3 | Lgals3 | 3958 | 16854 |
| MAOB | Maob | 4129 | 109731 |
| IST1 | Ist1 | 9798 | 71955 |
| UTRN | Utrn | 7402 | 22288 |
| BTD | Btd | 686 | 26363 |
| RAB2A | Rab2a | 5862 | 59021 |
| EML2 | Eml2 | 24139 | 72205 |
| SEPT10 | Sept10 | 151011 | 103080 |
| PNPT1 | Pnpt1 | 87178 | 71701 |
| PLAA | Plaa | 9373 | 18786 |
| WBP2 | Wbp2 | 23558 | 22378 |
| CDKN2AIPNL | Cdkn2aipnl | 91368 | 52626 |
| MAP7D1 | Map7d1 | 55700 | 245877 |
| APOA2 | Apoa2 | 336 | 11807 |
| NDUFB4 | Ndufb4 | 4710 | 68194 |
| NDUFB4 | Gm3244 | 4710 | 100041273 |
| NDUFB4 | Gm3873 | 4710 | 100042503 |
| MVD | Mvd | 4597 | 192156 |
| GPX4 | Gpx4 | 2879 | 625249 |
| BUB3 | Bub3 | 9184 | 12237 |
| RAD23B | Rad23b | 5887 | 19359 |
| EHD2 | Ehd2 | 30846 | 259300 |
| PLBD2 | Plbd2 | 196463 | 71772 |
| TLN1 | Tln1 | 7094 | 21894 |
| PHB2 | Phb2 | 11331 | 12034 |
| KPNA4 | Kpna4 | 3840 | 16649 |
| PNP | Pnp2 | 4860 | 667034 |
| PNP | Pnp | 4860 | 18950 |
| LTBP4 | Ltbp4 | 8425 | 108075 |
| ACO1 | Aco1 | 48 | 11428 |
| KCTD12 | Kctd12 | 115207 | 239217 |
| PSAT1 | Psat1 | 29968 | 107272 |
| COPS5 | Cops5 | 10987 | 26754 |
| GNS | Gns | 2799 | 75612 |
| PALM2 | Palm2 | 114299 | 242481 |
| PGM2 | Pgm1 | 55276 | 66681 |
| SNRPD1 | Snrpd1 | 6632 | 20641 |
| CDKN2C | Cdkn2c | 1031 | 12580 |
| ACLY | Acly | 47 | 104112 |
| PALM | Palm | 5064 | 18483 |
| ABCA8 | Abca8b | 10351 | 27404 |
| TXNL1 | Txnl1 | 9352 | 53382 |
| DYNLL2 | Dynll2 | 140735 | 68097 |
| IDE | Ide | 3416 | 15925 |
| CD36 | Cd36 | 948 | 12491 |
| CELF2 | Celf2 | 10659 | 14007 |
| VTI1B | Vti1b | 10490 | 53612 |
| AP2A2 | Ap2a2 | 161 | 11772 |
| HIST1H2BL | Hist1h2bj | 8340 | 319183 |
| FDPS | Fdps | 2224 | 110196 |
| COPG1 | Copg1 | 22820 | 54161 |
| HK2 | Hk2 | 3099 | 15277 |
| ISOC1 | Isoc1 | 51015 | 66307 |
| OXSR1 | Oxsr1 | 9943 | 108737 |
| HBB | Hbb-bs | 3043 | 100503605 |
| HBB | Hbb-bt | 3043 | 101488143 |
| MTPN | Mtpn | 136319 | 14489 |
| FN3KRP | Fn3krp | 79672 | 238024 |
| MFAP4 | Mfap4 | 4239 | 76293 |
| AKR1C1 | Akr1c21 | 1645 | 77337 |
| KIF5B | Kif5b | 3799 | 16573 |
| HPX | Hpx | 3263 | 15458 |
| MARCKSL1 | Marcksl1 | 65108 | 17357 |
| ENOPH1 | Enoph1 | 58478 | 67870 |
| UCHL1 | Uchl1 | 7345 | 22223 |
| SEPT11 | Sept11 | 55752 | 52398 |
| CAMK1 | Camk1 | 8536 | 52163 |
| FHL1 | Fhl1 | 2273 | 14199 |
| CSAD | Csad | 51380 | 246277 |
| IQGAP2 | Iqgap2 | 10788 | 544963 |
| GPT | Gpt | 2875 | 76282 |
| VAMP2 | Vamp2 | 6844 | 22318 |
| ACSL1 | Acsl1 | 2180 | 14081 |
| FLNA | Flna | 2316 | 192176 |
| PGGT1B | Pggt1b | 5229 | 225467 |
| HIST1H4A | Hist1h4d | 8359 | 319156 |
| CHCHD2 | Gm13202 | 51142 | 433806 |
| CHCHD2 | Chchd2 | 51142 | 14004 |
| PCOLCE | Pcolce | 5118 | 18542 |
| CSTB | Cstb | 1476 | 13014 |
| AIFM2 | Aifm2 | 84883 | 71361 |
| PEBP1 | Pebp1 | 5037 | 23980 |
| GABPA | Gabpa | 2551 | 14390 |
| CCT5 | Cct5 | 22948 | 12465 |
| ARL6IP1 | Arl6ip1 | 23204 | 54208 |
| FIS1 | Fis1 | 51024 | 66437 |
| NENF | Nenf | 29937 | 66208 |
| TMED10 | Tmed10 | 10972 | 68581 |
| TMED10 | Gm4024 | 10972 | 100042773 |
| MYH9 | Myh9 | 4627 | 17886 |
| SACM1L | Sacm1l | 22908 | 83493 |
| ACADL | Acadl | 33 | 11363 |
| ACP1 | Acp1 | 52 | 11431 |
| ABRACL | Abracl | 58527 | 73112 |
| RNPEP | Rnpep | 6051 | 215615 |
| GLO1 | Glo1 | 2739 | 109801 |
| AQP1 | Aqp1 | 358 | 11826 |
| GPD1 | Gpd1 | 2819 | 14555 |
| PPM1B | Ppm1b | 5495 | 19043 |
| NMT1 | Nmt1 | 4836 | 18107 |
| NPEPPS | Npepps | 9520 | 19155 |
| LGALS1 | Lgals1 | 3956 | 16852 |
| EIF2S3 | Eif2s3x | 1968 | 26905 |
| EFHD1 | Efhd1 | 80303 | 98363 |
| GSR | Gsr | 2936 | 14782 |
| FAIM | Faim | 55179 | 23873 |
| ALDH9A1 | Aldh9a1 | 223 | 56752 |
| PPP2CB | Ppp2cb | 5516 | 19053 |
| ILVBL | Ilvbl | 10994 | 216136 |
| STAT1 | Stat1 | 6772 | 20846 |
| RRM2B | Rrm2b | 50484 | 382985 |
| CALR | Calr | 811 | 12317 |
| PPIL1 | Ppil1 | 51645 | 68816 |
| AP2A1 | Ap2a1 | 160 | 11771 |
| BPGM | Bpgm | 669 | 12183 |
| OGDH | Ogdh | 4967 | 18293 |
| TATDN1 | Tatdn1 | 83940 | 69694 |
| TXNRD1 | Txnrd1 | 7296 | 50493 |
| PDIA6 | Pdia6 | 10130 | 71853 |
| KDSR | Kdsr | 2531 | 70750 |
| RPL10A | Rpl10a | 4736 | 19896 |
| ZYX | Zyx | 7791 | 22793 |
| GGT5 | Ggt5 | 2687 | 23887 |
| PFDN2 | Pfdn2 | 5202 | 18637 |
| NID1 | Nid1 | 4811 | 18073 |
| LMCD1 | Lmcd1 | 29995 | 30937 |
| GOLGB1 | Golgb1 | 2804 | 224139 |
| PNMA2 | Pnma2 | 10687 | 239157 |
| DHX9 | Dhx9 | 1660 | 13211 |
| PPIL3 | Ppil3 | 53938 | 70225 |
| HSP90AA1 | Hsp90aa1 | 3320 | 15519 |
| RPS15A | Rps15a | 6210 | 267019 |
| ARPC1A | Arpc1a | 10552 | 56443 |
| VWF | Vwf | 7450 | 22371 |
| PLIN3 | Plin3 | 10226 | 66905 |
| GLYAT | Glyat | 10249 | 107146 |
| CKM | Ckm | 1158 | 12715 |
| STXBP3 | Stxbp3a | 6814 | 20912 |
| LACTB2 | Lactb2 | 51110 | 212442 |
| ADRM1 | Adrm1 | 11047 | 56436 |
| IFI16 | Ifi204 | 3428 | 15951 |
| IFI16 | BC094916 | 3428 | 545384 |
| IFI16 | Pyhin1 | 3428 | 236312 |
| IFI16 | Ifi203 | 3428 | 15950 |
| IFI16 | Gm16340 | 3428 | 100504287 |
| IFI16 | Mndal | 3428 | 100040462 |
| IFI16 | Ifi205 | 3428 | 226695 |
| IFI16 | Mnda | 3428 | 381308 |
| IFI16 | AI607873 | 3428 | 226691 |
| IFI16 | LOC102639543 | 3428 | 102639543 |
| IFI16 | Pydc3 | 3428 | 100033459 |
| IFI16 | Pydc4 | 3428 | 623121 |
| CAP2 | Cap2 | 10486 | 67252 |
| PGP | Pgp | 283871 | 67078 |
| PDIA3 | Pdia3 | 2923 | 14827 |
| PFDN4 | Pfdn4 | 5203 | 109054 |
| HEXA | Hexa | 3073 | 15211 |
| PSMB6 | Psmb6 | 5694 | 19175 |
| EEF2 | Eef2 | 1938 | 13629 |
| FAHD1 | Fahd1 | 81889 | 68636 |
| SCFD1 | Scfd1 | 23256 | 76983 |
| HIBCH | Hibch | 26275 | 227095 |
| SCARB2 | Scarb2 | 950 | 12492 |
| FTO | Fto | 79068 | 26383 |
| SORT1 | Sort1 | 6272 | 20661 |
| LRP1 | Lrp1 | 4035 | 16971 |
| LANCL2 | Lancl2 | 55915 | 71835 |
| TMX1 | Tmx1 | 81542 | 72736 |
| PCBD1 | Pcbd1 | 5092 | 13180 |
| SPTBN1 | Sptbn1 | 6711 | 20742 |
| COMMD5 | Commd5 | 28991 | 66398 |
| EPB41L2 | Epb4.1l2 | 2037 | 13822 |
| ACYP2 | Acyp2 | 98 | 75572 |
| FN1 | Fn1 | 2335 | 14268 |
| NACA | Naca | 4666 | 17938 |
| PPP1R12A | Ppp1r12a | 4659 | 17931 |
| SPTAN1 | Sptan1 | 6709 | 20740 |
| ERC1 | Erc1 | 23085 | 111173 |
| HNRNPA2B1 | Hnrnpa2b1 | 3181 | 53379 |
| CCT2 | Cct2 | 10576 | 12461 |
| PCBP1 | Pcbp1 | 5093 | 23983 |
| MDH2 | Mdh2 | 4191 | 17448 |
| EML4 | Eml4 | 27436 | 78798 |
| UQCRQ | Uqcrq | 27089 | 22272 |
| FTL | Ftl1 | 2512 | 14325 |
| FTL | Gm20746 | 2512 | 434624 |
| DCTN1 | Dctn1 | 1639 | 13191 |
| ECHS1 | Echs1 | 1892 | 93747 |
| TRAPPC3 | Trappc3 | 27095 | 27096 |
| ENO2 | Eno2 | 2026 | 13807 |
| CD81 | Cd81 | 975 | 12520 |
| PICALM | Picalm | 8301 | 233489 |
| CDC37 | Cdc37 | 11140 | 12539 |
| YWHAE | Ywhae | 7531 | 22627 |
| SAFB | Safb | 6294 | 224903 |
| STAT6 | Stat6 | 6778 | 20852 |
| FAH | Fah | 2184 | 14085 |
| PSMB4 | Psmb4 | 5692 | 19172 |
| PPA2 | Ppa2 | 27068 | 74776 |
| GLA | Gla | 2717 | 11605 |
| PRELP | Prelp | 5549 | 116847 |
| LAMP1 | Lamp1 | 3916 | 16783 |
| TBCD | Tbcd | 6904 | 108903 |
| CALB2 | Calb2 | 794 | 12308 |
| PAICS | Paics | 10606 | 67054 |
| NDUFA13 | Ndufa13 | 51079 | 67184 |
| MAOA | Maoa | 4128 | 17161 |
| NDUFAB1 | Ndufab1 | 4706 | 70316 |
| NDUFAB1 | LOC102634451 | 4706 | 102634451 |
| ATP2A2 | Atp2a2 | 488 | 11938 |
| CAP1 | Cap1 | 10487 | 12331 |
| PPCS | Ppcs | 79717 | 106564 |
| CLTB | Cltb | 1212 | 74325 |
| HADHB | Hadhb | 3032 | 231086 |
| APPL1 | Appl1 | 26060 | 72993 |
| PREP | Prep | 5550 | 19072 |
| PRDX1 | Prdx1 | 5052 | 18477 |
| COPS6 | Cops6 | 10980 | 26893 |
| UTS2 | Uts2 | 10911 | 24111 |
| ALDH1L1 | Aldh1l1 | 10840 | 107747 |
| COPS4 | Cops4 | 51138 | 26891 |
| MTAP | Mtap | 4507 | 66902 |
| SNX2 | Snx2 | 6643 | 67804 |
| MVP | Mvp | 9961 | 78388 |
| SPON1 | Spon1 | 10418 | 233744 |
| CYFIP1 | Cyfip1 | 23191 | 20430 |
| PRPS1 | Prps1 | 5631 | 19139 |
| TKT | Tkt | 7086 | 21881 |
| ITGAM | Itgam | 3684 | 16409 |
| PPP3R1 | Ppp3r1 | 5534 | 19058 |
| CKMT2 | Ckmt2 | 1160 | 76722 |
| ZNF428 | Zfp428 | 126299 | 232969 |
| TXN2 | Txn2 | 25828 | 56551 |
| TIMM8A | Timm8a1 | 1678 | 30058 |
| PDLIM1 | Pdlim1 | 9124 | 54132 |
| COPZ2 | Copz2 | 51226 | 56358 |
| LAMTOR3 | Lamtor3 | 8649 | 56692 |
| ERP29 | Erp29 | 10961 | 67397 |
| FSCN1 | Fscn1 | 6624 | 14086 |
| SPRYD4 | Spryd4 | 283377 | 66701 |
| COX6B1 | Cox6b1 | 1340 | 110323 |
| FH | Fh1 | 2271 | 14194 |
| ARPC5 | Arpc5 | 10092 | 67771 |
| ACOT2 | Acot2 | 10965 | 171210 |
| COPS3 | Cops3 | 8533 | 26572 |
| MAP4 | Map4 | 4134 | 17758 |
| MYLK | Mylk | 4638 | 107589 |
| ADPRHL2 | Adprhl2 | 54936 | 100206 |
| DLAT | Dlat | 1737 | 235339 |
| SUCLG2 | Suclg2 | 8801 | 20917 |
| SLC19A3 | Slc19a3 | 80704 | 80721 |
| EEF1E1 | Eef1e1 | 9521 | 66143 |
| AIMP1 | Aimp1 | 9255 | 13722 |
| SMU1 | Smu1 | 55234 | 74255 |
| TNNC2 | Tnnc2 | 7125 | 21925 |
| IDH3G | Idh3g | 3421 | 15929 |
| ATP5J | Atp5j | 522 | 11957 |
| MRC2 | Mrc2 | 9902 | 17534 |
| HNRNPL | Hnrnpl | 3191 | 15388 |
| VPS45 | Vps45 | 11311 | 22365 |
| PPP2R2A | Ppp2r2a | 5520 | 71978 |
| CRK | Crk | 1398 | 12928 |
| AIF1 | Aif1 | 199 | 11629 |
| IL18R1 | Il18r1 | 8809 | 16182 |
| SND1 | Snd1 | 27044 | 56463 |
| STAM | Stam | 8027 | 20844 |
| ACAD9 | Acad9 | 28976 | 229211 |
| ERP44 | Erp44 | 23071 | 76299 |
| TNS1 | Tns1 | 7145 | 21961 |
| AAMDC | Aamdc | 28971 | 66273 |
| PAFAH1B1 | Pafah1b1 | 5048 | 18472 |
| PPT1 | Ppt1 | 5538 | 19063 |
| DPP3 | Dpp3 | 10072 | 75221 |
| COL15A1 | Col15a1 | 1306 | 12819 |
| RABGGTA | Rabggta | 5875 | 56187 |
| DFFA | Dffa | 1676 | 13347 |
| RAB21 | Rab21 | 23011 | 216344 |
| YES1 | Yes1 | 7525 | 22612 |
| IBA57 | Iba57 | 200205 | 216792 |
| BLOC1S2 | Bloc1s2 | 282991 | 73689 |
| PTPRM | Ptprm | 5797 | 19274 |
| EIF3L | Eif3l | 51386 | 223691 |
| ADAR | Adar | 103 | 56417 |
| SLC25A11 | Slc25a11 | 8402 | 67863 |
| GBP2 | Gbp2 | 2634 | 14469 |
| UBA52 | Gm7866 | 7311 | 665964 |
| TSNAX | Tsnax | 7257 | 53424 |
| GLIPR2 | Glipr2 | 152007 | 384009 |
| PPA1 | Ppa1 | 5464 | 67895 |
| MAP1B | Map1b | 4131 | 17755 |
| STRAP | Strap | 11171 | 20901 |
| HNRNPH1 | Hnrnph1 | 3187 | 59013 |
| SNRPN | Snrpn | 6638 | 20646 |
| ADD1 | Add1 | 118 | 11518 |
| MLEC | Mlec | 9761 | 109154 |
| ACTC1 | Actc1 | 70 | 11464 |
| ACAD8 | Acad8 | 27034 | 66948 |
| TPT1 | Tpt1 | 7178 | 22070 |
| PDIA4 | Pdia4 | 9601 | 12304 |
| ASPH | Asph | 444 | 65973 |
| PEA15 | Pea15a | 8682 | 18611 |
| NEDD8 | Nedd8 | 4738 | 18002 |
| PSMC3 | Psmc3 | 5702 | 19182 |
| CCDC43 | Ccdc43 | 124808 | 52715 |
| PTK2 | Ptk2 | 5747 | 14083 |
| HIP1 | Hip1 | 3092 | 215114 |
| HUWE1 | Huwe1 | 10075 | 59026 |
| ECHDC2 | Echdc2 | 55268 | 52430 |
| PC | Pcx | 5091 | 18563 |
| FSTL1 | Fstl1 | 11167 | 14314 |
| NIF3L1 | Nif3l1 | 60491 | 65102 |
| SDHB | Sdhb | 6390 | 67680 |
| CCS | Ccs | 9973 | 12460 |
| TRAPPC4 | Trappc4 | 51399 | 60409 |
| C1QB | C1qb | 713 | 12260 |
| LAMP2 | Lamp2 | 3920 | 16784 |
| IMPA1 | Impa1 | 3612 | 55980 |
| AKR1C3 | Akr1c18 | 8644 | 105349 |
| CPSF6 | Cpsf6 | 11052 | 432508 |
| CLPP | Clpp | 8192 | 53895 |
| NUDT21 | Nudt21 | 11051 | 68219 |
| C21orf33 | D10Jhu81e | 8209 | 28295 |
| SF3B2 | Sf3b2 | 10992 | 319322 |
| PARK7 | Park7 | 11315 | 57320 |
| CBR1 | Cbr1 | 873 | 12408 |
| UBE2V1 | Ube2v1 | 7335 | 66589 |
| FKBP1A | Fkbp1a | 2280 | 14225 |
| RPA2 | Rpa2 | 6118 | 19891 |
| TOM1 | Tom1 | 10043 | 21968 |
| DCXR | Dcxr | 51181 | 67880 |
| LACTB | Lactb | 114294 | 80907 |
| CD14 | Cd14 | 929 | 12475 |
| MARS | Mars | 4141 | 216443 |
| CSE1L | Cse1l | 1434 | 110750 |
| ATL3 | Atl3 | 25923 | 109168 |
| PRCP | Prcp | 5547 | 72461 |
| S100A10 | S100a10 | 6281 | 20194 |
| SNRPA1 | Snrpa1 | 6627 | 68981 |
| TWF1 | Twf1 | 5756 | 19230 |
| SUCLA2 | Sucla2 | 8803 | 20916 |
| MAT2A | Mat2a | 4144 | 232087 |
| NDUFB10 | Ndufb10 | 4716 | 68342 |
| RPL7 | Rpl7 | 6129 | 19989 |
| NME1 | Nme1 | 4830 | 18102 |
| FARSB | Farsb | 10056 | 23874 |
| ATP5J2 | Atp5j2 | 9551 | 57423 |
| ITGB6 | Itgb6 | 3694 | 16420 |
| PSMD6 | Psmd6 | 9861 | 66413 |
| TRAPPC6B | Trappc6b | 122553 | 78232 |
| GEMIN5 | Gemin5 | 25929 | 216766 |
| CDV3 | Cdv3 | 55573 | 321022 |
| CDV3 | LOC102639170 | 55573 | 102639170 |
| CLIC6 | Clic6 | 54102 | 209195 |
| RHOA | Rhoa | 387 | 11848 |
| TXNDC5 | Txndc5 | 81567 | 105245 |
| MPST | Mpst | 4357 | 246221 |
| PTP4A2 | Ptp4a2 | 8073 | 19244 |
| CAB39 | Cab39 | 51719 | 12283 |
| RTCB | Rtcb | 51493 | 28088 |
| ABI1 | Abi1 | 10006 | 11308 |
| ECI1 | Eci1 | 1632 | 13177 |
| EIF3D | Eif3d | 8664 | 55944 |
| CD93 | Cd93 | 22918 | 17064 |
| PTBP1 | Ptbp1 | 5725 | 19205 |
| EEF1B2 | Eef1b2 | 1933 | 55949 |
| AIMP2 | Aimp2 | 7965 | 231872 |
| COL6A1 | Col6a1 | 1291 | 12833 |
| NUDCD3 | Nudcd3 | 23386 | 209586 |
| ZFAND1 | Zfand1 | 79752 | 66361 |
| ITIH5 | Itih5 | 80760 | 209378 |
| HS1BP3 | Hs1bp3 | 64342 | 58240 |
| EIF3K | Eif3k | 27335 | 73830 |
| ME1 | Me1 | 4199 | 17436 |
| HNRNPD | Hnrnpd | 3184 | 11991 |
| GMFB | Gmfb | 2764 | 63985 |
| CCT4 | Cct4 | 10575 | 12464 |
| RPS3 | Rps3 | 6188 | 27050 |
| RCC2 | Rcc2 | 55920 | 108911 |
| CNDP2 | Cndp2 | 55748 | 66054 |
| MTHFD1 | Mthfd1 | 4522 | 108156 |
| BAG6 | Bag6 | 7917 | 224727 |
| RRAS2 | Rras2 | 22800 | 66922 |
| TIMM9 | Timm9 | 26520 | 30056 |
| VDAC2 | Vdac2 | 7417 | 22334 |
| RMDN3 | Rmdn3 | 55177 | 67809 |
| SPTLC3 | Sptlc3 | 55304 | 228677 |
| CST3 | Cst3 | 1471 | 13010 |
| ARL6IP5 | Arl6ip5 | 10550 | 65106 |
| CAT | Cat | 847 | 12359 |
| SARS2 | Sars2 | 54938 | 71984 |
| ETFA | Etfa | 2108 | 110842 |
| CLINT1 | Clint1 | 9685 | 216705 |
| LYVE1 | Lyve1 | 10894 | 114332 |
| SPAG9 | Spag9 | 9043 | 70834 |
| FERMT3 | Fermt3 | 83706 | 108101 |
| SNRNP70 | Snrnp70 | 6625 | 20637 |
| LSM2 | Lsm2 | 57819 | 27756 |
| QDPR | Qdpr | 5860 | 110391 |
| DHRS7 | Dhrs7 | 51635 | 66375 |
| DMGDH | Dmgdh | 29958 | 74129 |
| EIF3C | Eif3c | 8663 | 56347 |
| EIF3M | Eif3m | 10480 | 98221 |
| ASS1 | Ass1 | 445 | 11898 |
| PANK4 | Pank4 | 55229 | 269614 |
| PGK1 | Pgk1 | 5230 | 18655 |
| EIF3I | Eif3i | 8668 | 54709 |
| SHMT1 | Shmt1 | 6470 | 20425 |
| SLC25A4 | Slc25a4 | 291 | 11739 |
| APEH | Apeh | 327 | 235606 |
| LONP1 | Lonp1 | 9361 | 74142 |
| BRCC3 | Brcc3 | 79184 | 210766 |
| TXNDC12 | Txndc12 | 51060 | 66073 |
| EPB41 | Epb4.1 | 2035 | 269587 |
| ARHGDIB | Arhgdib | 397 | 11857 |
| ARHGAP17 | Arhgap17 | 55114 | 70497 |
| BTF3L4 | Btf3l4 | 91408 | 70533 |
| PEX19 | Pex19 | 5824 | 19298 |
| ETHE1 | Ethe1 | 23474 | 66071 |
| NDUFB3 | Ndufb3 | 4709 | 66495 |
| SYNCRIP | Syncrip | 10492 | 56403 |
| GGCT | Ggct | 79017 | 110175 |
| NAGK | Nagk | 55577 | 56174 |
| IMMT | Immt | 10989 | 76614 |
| SCAF4 | Scaf4 | 57466 | 224432 |
| PRPSAP2 | Prpsap2 | 5636 | 212627 |
| ACTR10 | Actr10 | 55860 | 56444 |
| VDAC3 | Vdac3 | 7419 | 22335 |
| PFKL | Pfkl | 5211 | 18641 |
| UBE2L3 | Ube2l3 | 7332 | 22195 |
| SUCLG1 | Suclg1 | 8802 | 56451 |
| CMPK1 | Cmpk1 | 51727 | 66588 |
| HNRNPF | Hnrnpf | 3185 | 98758 |
| CCDC58 | Ccdc58 | 131076 | 381045 |
| LGALS3BP | Lgals3bp | 3959 | 19039 |
| ADD3 | Add3 | 120 | 27360 |
| M6PR | M6pr | 4074 | 17113 |
| LYPLA2 | Lypla2 | 11313 | 26394 |
| PRDX3 | Prdx3 | 10935 | 11757 |
| ILK | Ilk | 3611 | 16202 |
| TPPP3 | Tppp3 | 51673 | 67971 |
| LUC7L2 | Luc7l2 | 51631 | 192196 |
| COL18A1 | Col18a1 | 80781 | 12822 |
| MYL6 | Myl6 | 4637 | 17904 |
| SWAP70 | Swap70 | 23075 | 20947 |
| CUL4A | Cul4a | 8451 | 99375 |
| HDDC3 | Hddc3 | 374659 | 68695 |
| EPB41L3 | Epb4.1l3 | 23136 | 13823 |
| YWHAH | Ywhah | 7533 | 22629 |
| MCEE | Mcee | 84693 | 73724 |
| PPP1R18 | Ppp1r18 | 170954 | 76448 |
| ALDH16A1 | Aldh16a1 | 126133 | 69748 |
| BABAM1 | Babam1 | 29086 | 68251 |
| PON1 | Pon1 | 5444 | 18979 |
| GIT2 | Git2 | 9815 | 26431 |
| STRN3 | Strn3 | 29966 | 94186 |
| RAP1B | Rap1b | 5908 | 215449 |
| RABL6 | Rabl6 | 55684 | 227624 |
| APEX1 | Apex1 | 328 | 11792 |
| EDC4 | Edc4 | 23644 | 234699 |
| MIF | Mif | 4282 | 17319 |
| BZW2 | Bzw2 | 28969 | 66912 |
| PML | Pml | 5371 | 18854 |
| AP2M1 | Ap2m1 | 1173 | 11773 |
| HNRNPM | Hnrnpm | 4670 | 76936 |
| MYOF | Myof | 26509 | 226101 |
| UQCRH | Gm9763 | 7388 | 100042918 |
| COX7A2 | Cox7a2 | 1347 | 12866 |
| NPEPL1 | Npepl1 | 79716 | 228961 |
| PHYHD1 | Phyhd1 | 254295 | 227696 |
| TROVE2 | Trove2 | 6738 | 20822 |
| GOLGA4 | Golga4 | 2803 | 54214 |
| F13A1 | F13a1 | 2162 | 74145 |
| METTL7A | Mettl7a1 | 25840 | 70152 |
| ACSF2 | Acsf2 | 80221 | 264895 |
| ABLIM1 | Ablim1 | 3983 | 226251 |
| HGS | Hgs | 9146 | 15239 |
| MYO1C | Myo1c | 4641 | 17913 |
| SLC9A3R1 | Slc9a3r1 | 9368 | 26941 |
| SF3A1 | Sf3a1 | 10291 | 67465 |
| PCYT2 | Pcyt2 | 5833 | 68671 |
| MECP2 | Mecp2 | 4204 | 17257 |
| PDHX | Pdhx | 8050 | 27402 |
| TANGO2 | Tango2 | 128989 | 27883 |
| PLEC | Plec | 5339 | 18810 |
| ANXA7 | Anxa7 | 310 | 11750 |
| PPME1 | Ppme1 | 51400 | 72590 |
| DCAF8 | Dcaf8 | 50717 | 98193 |
| TWF2 | Twf2 | 11344 | 23999 |
| ACOX1 | Acox1 | 51 | 11430 |
| PLD3 | Pld3 | 23646 | 18807 |
| NAGA | Naga | 4668 | 17939 |
| WDR1 | Wdr1 | 9948 | 22388 |
| NQO2 | Nqo2 | 4835 | 18105 |
| LDHA | Ldha | 3939 | 16828 |
| PDCD6 | Pdcd6 | 10016 | 18570 |
| HINT1 | Hint1 | 3094 | 15254 |
| IAH1 | Iah1 | 285148 | 67732 |
| GPS1 | Gps1 | 2873 | 209318 |
| S100A9 | S100a9 | 6280 | 20202 |
| HSPA2 | Hspa2 | 3306 | 15512 |
| PSAP | Psap | 5660 | 19156 |
| ANXA2 | Anxa2 | 302 | 12306 |
| EFEMP1 | Efemp1 | 2202 | 216616 |
| MCAM | Mcam | 4162 | 84004 |
| PSMA7 | Psma7 | 5688 | 26444 |
| TMPO | Tmpo | 7112 | 21917 |
| TTC38 | Ttc38 | 55020 | 239570 |
| PSMD2 | Psmd2 | 5708 | 21762 |
| U2AF2 | U2af2 | 11338 | 22185 |
| PGD | Pgd | 5226 | 110208 |
| LIMCH1 | Limch1 | 22998 | 77569 |
| PRMT1 | Prmt1 | 3276 | 15469 |
| RANBP3 | Ranbp3 | 8498 | 71810 |
| CTNNA1 | Ctnna1 | 1495 | 12385 |
| FAM114A1 | Fam114a1 | 92689 | 68303 |
| COPS8 | Cops8 | 10920 | 108679 |
| RTN4 | Rtn4 | 57142 | 68585 |
| RPLP2 | Rplp2 | 6181 | 67186 |
| FGG | Fgg | 2266 | 99571 |
| PDPR | Pdpr | 55066 | 319518 |
| CMAS | Cmas | 55907 | 12764 |
| GNPDA1 | Gnpda1 | 10007 | 26384 |
| ECHDC1 | Echdc1 | 55862 | 52665 |
| SBDS | Sbds | 51119 | 66711 |
| RBP7 | Rbp7 | 116362 | 63954 |
| FUBP1 | Fubp1 | 8880 | 51886 |
| UAP1 | Uap1 | 6675 | 107652 |
| NIPSNAP3A | Nipsnap3b | 25934 | 66536 |
| TMLHE | Tmlhe | 55217 | 192289 |
| VBP1 | Vbp1 | 7411 | 22327 |
| LASP1 | Lasp1 | 3927 | 16796 |
| ME3 | Me3 | 10873 | 109264 |
| SCRN3 | Scrn3 | 79634 | 74616 |
| STIP1 | Stip1 | 10963 | 20867 |
| UBE2M | Ube2m | 9040 | 22192 |
| DPP9 | Dpp9 | 91039 | 224897 |
| PGAM1 | Pgam1 | 5223 | 18648 |
| CAPZB | Capzb | 832 | 12345 |
| GNG2 | Gng2 | 54331 | 14702 |
| CLTA | Clta | 1211 | 12757 |
| UQCRB | Uqcrb | 7381 | 67530 |
| SNCA | Snca | 6622 | 20617 |
| GFPT1 | Gfpt1 | 2673 | 14583 |
| WDR45B | Wdr45b | 56270 | 66840 |
| TBCB | Tbcb | 1155 | 66411 |
| SPARC | Sparc | 6678 | 20692 |
| PFDN6 | H2-Ke2 | 10471 | 14976 |
| CA1 | Car1 | 759 | 12346 |
| RPS2 | Rps2 | 6187 | 16898 |
| PTGR2 | Ptgr2 | 145482 | 77219 |
| CTSG | Ctsg | 1511 | 13035 |
| CSDE1 | Csde1 | 7812 | 229663 |
| S100A8 | S100a8 | 6279 | 20201 |
| PPM1F | Ppm1f | 9647 | 68606 |
| EML3 | Eml3 | 256364 | 225898 |
| GNPDA2 | Gnpda2 | 132789 | 67980 |
| MGST2 | Mgst2 | 4258 | 211666 |
| PURA | Pura | 5813 | 19290 |
| NID2 | Nid2 | 22795 | 18074 |
| CTSA | Ctsa | 5476 | 19025 |
| RNH1 | Rnh1 | 6050 | 107702 |
| CYB5A | Cyb5 | 1528 | 109672 |
| BLMH | Blmh | 642 | 104184 |
| LRRC47 | Lrrc47 | 57470 | 72946 |
| DHRS11 | Dhrs11 | 79154 | 192970 |
| HSD17B12 | Hsd17b12 | 51144 | 56348 |
| ALDH1B1 | Aldh1b1 | 219 | 72535 |
| NUDT2 | Nudt2 | 318 | 66401 |
| PPP1R14B | Ppp1r14b | 26472 | 18938 |
| PKM | Pkm | 5315 | 18746 |
| PALLD | Palld | 23022 | 72333 |
| ARHGEF6 | Arhgef6 | 9459 | 73341 |
| LIN7C | Lin7c | 55327 | 22343 |
| PLIN4 | Plin4 | 729359 | 57435 |
| NSFL1C | Nsfl1c | 55968 | 386649 |
| COL4A2 | Col4a2 | 1284 | 12827 |
| MFAP5 | Mfap5 | 8076 | 50530 |
| SYNGR2 | Syngr2 | 9144 | 20973 |
| GPX3 | Gpx3 | 2878 | 14778 |
| TARS | Tars | 6897 | 110960 |
| NUTF2 | Nutf2 | 10204 | 68051 |
| NUTF2 | Gm10349 | 10204 | 621832 |
| MAN2C1 | Man2c1 | 4123 | 73744 |
| HADHA | Hadha | 3030 | 97212 |
| C1QBP | C1qbp | 708 | 12261 |
| TOLLIP | Tollip | 54472 | 54473 |
| ASL | Asl | 435 | 109900 |
| ARPC4 | Arpc4 | 10093 | 68089 |
| OXR1 | Oxr1 | 55074 | 170719 |
| RUVBL1 | Ruvbl1 | 8607 | 56505 |
| CA3 | Car3 | 761 | 12350 |
| ME2 | Me2 | 4200 | 107029 |
| POR | Por | 5447 | 18984 |
| FKBP2 | Fkbp2 | 2286 | 14227 |
| AK3 | Ak3 | 50808 | 56248 |
| AP1M1 | Ap1m1 | 8907 | 11767 |
| ATOX1 | Atox1 | 475 | 11927 |
| ACTR1A | Actr1a | 10121 | 54130 |
| HK1 | Hk1 | 3098 | 15275 |
| PUF60 | Puf60 | 22827 | 67959 |
| LAMB1 | Lamb1 | 3912 | 16777 |
| SOD2 | Sod2 | 6648 | 20656 |
| API5 | Api5 | 8539 | 11800 |
| NDUFS7 | Ndufs7 | 374291 | 75406 |
| TJP2 | Tjp2 | 9414 | 21873 |
| PLCD1 | Plcd1 | 5333 | 18799 |
| PROS1 | Pros1 | 5627 | 19128 |
| COPB1 | Copb1 | 1315 | 70349 |
| YWHAQ | Ywhaq | 10971 | 22630 |
| YWHAQ | LOC102634437 | 10971 | 102634437 |
| CSRP1 | Csrp1 | 1465 | 13007 |
| UBA2 | Uba2 | 10054 | 50995 |
| PEPD | Pepd | 5184 | 18624 |
| BPNT1 | Bpnt1 | 10380 | 23827 |
| MYL1 | Myl1 | 4632 | 17901 |
| S100A16 | S100a16 | 140576 | 67860 |
| PMPCA | Pmpca | 23203 | 66865 |
| EPS15L1 | Eps15l1 | 58513 | 13859 |
| NUMA1 | Numa1 | 4926 | 101706 |
| ADIPOQ | Adipoq | 9370 | 11450 |
| GALK1 | Galk1 | 2584 | 14635 |
| ILF2 | Ilf2 | 3608 | 67781 |
| GDI1 | Gdi1 | 2664 | 14567 |
| ANPEP | Anpep | 290 | 16790 |
| CFB | Cfb | 629 | 14962 |
| HDDC2 | Hddc2 | 51020 | 69692 |
| ANXA1 | Anxa1 | 301 | 16952 |
| SF3B6 | 0610009D07Rik | 51639 | 66055 |
| CRYAB | Cryab | 1410 | 12955 |
| UFM1 | Ufm1 | 51569 | 67890 |
| SRP9 | Srp9 | 6726 | 27058 |
| TRIP10 | Trip10 | 9322 | 106628 |
| SEC22B | Sec22b | 9554 | 20333 |
| ALDH5A1 | Aldh5a1 | 7915 | 214579 |
| PFDN5 | Pfdn5 | 5204 | 56612 |
| ESD | Esd | 2098 | 13885 |
| ESD | Gm2904 | 2098 | 100040682 |
| RETSAT | Retsat | 54884 | 67442 |
| NASP | Nasp | 4678 | 50927 |
| FBP1 | Fbp1 | 2203 | 14121 |
| PIR | Pir | 8544 | 69656 |
| PCK2 | Pck2 | 5106 | 74551 |
| HDHD2 | Hdhd2 | 84064 | 76987 |
| NUDT16 | Nudt16 | 131870 | 75686 |
| AKR7A2 | Akr7a5 | 8574 | 110198 |
| POFUT1 | Pofut1 | 23509 | 140484 |
| DNPH1 | Dnph1 | 10591 | 381101 |
| BCAT2 | Bcat2 | 587 | 12036 |
| SET | Set | 6418 | 56086 |
| HSPB11 | Hspb11 | 51668 | 72938 |
| MARC1 | Marc1 | 64757 | 66112 |
| IMPDH2 | Gm15210 | 3615 | 100042069 |
| IMPDH2 | Impdh2 | 3615 | 23918 |
| CANX | Canx | 821 | 12330 |
| NDUFV1 | Ndufv1 | 4723 | 17995 |
| SLK | Slk | 9748 | 20874 |
| FMOD | Fmod | 2331 | 14264 |
| MDH1 | Mdh1 | 4190 | 17449 |
| RPN1 | Rpn1 | 6184 | 103963 |
| LSM8 | Lsm8 | 51691 | 76522 |
| UQCRC2 | Uqcrc2 | 7385 | 67003 |
| C12orf10 | Myg1 | 60314 | 60315 |
| DBNL | Dbnl | 28988 | 13169 |
| YWHAB | Ywhab | 7529 | 54401 |
| SLC25A5 | Slc25a5 | 292 | 11740 |
| ADSL | Adsl | 158 | 11564 |
| B2M | B2m | 567 | 12010 |
| EIF4A1 | Eif4a1 | 1973 | 13681 |
| HPRT1 | Hprt | 3251 | 15452 |
| TNKS1BP1 | Tnks1bp1 | 85456 | 228140 |
| AP3B1 | Ap3b1 | 8546 | 11774 |
| TRIM21 | Trim21 | 6737 | 20821 |
| ADA | Ada | 100 | 11486 |
| NDUFA9 | Ndufa9 | 4704 | 66108 |
| PHB | Phb | 5245 | 18673 |
| YWHAG | Ywhag | 7532 | 22628 |
| MB | Mb | 4151 | 17189 |
| PRDX4 | Prdx4 | 10549 | 53381 |
| TAF15 | Taf15 | 8148 | 70439 |
| EIF6 | Eif6 | 3692 | 16418 |
| COX6C | LOC102640946 | 1345 | 102640946 |
| TUFM | Tufm | 7284 | 233870 |
| PAIP1 | Paip1 | 10605 | 218693 |
| UROD | Urod | 7389 | 22275 |
| PLCB3 | Plcb3 | 5331 | 18797 |
| C1QC | C1qc | 714 | 12262 |
| EIF3B | Eif3b | 8662 | 27979 |
| CLIP1 | Clip1 | 6249 | 56430 |
| EIF2A | Eif2a | 83939 | 229317 |
| YBX1 | Ybx1 | 4904 | 22608 |
| PYGL | Pygl | 5836 | 110095 |
| ARPC1B | Arpc1b | 10095 | 11867 |
| CMBL | Cmbl | 134147 | 69574 |
| RAB1A | Rab1 | 5861 | 19324 |
| CFL1 | Cfl1 | 1072 | 12631 |
| AKAP1 | Akap1 | 8165 | 11640 |
| GLUD1 | Glud1 | 2746 | 14661 |
| COPB2 | Copb2 | 9276 | 50797 |
| GALM | Galm | 130589 | 319625 |
| UGP2 | Ugp2 | 7360 | 216558 |
| LETM1 | Letm1 | 3954 | 56384 |
| GTF2I | Gtf2i | 2969 | 14886 |
| ANXA11 | Anxa11 | 311 | 11744 |
| NARS | Nars | 4677 | 70223 |
| CAPZA1 | Capza1 | 829 | 12340 |
| NDUFS3 | Ndufs3 | 4722 | 68349 |
| NUDT5 | Nudt5 | 11164 | 53893 |
| CPPED1 | Cpped1 | 55313 | 223978 |
| PSME2 | Psme2 | 5721 | 19188 |
| SLC25A10 | Slc25a10 | 1468 | 27376 |
| NDRG1 | Ndrg1 | 10397 | 17988 |
| HSPA4 | Hspa4 | 3308 | 15525 |
| PPP3CB | Ppp3cb | 5532 | 19056 |
| TPR | Tpr | 7175 | 108989 |
| ASAH1 | Asah1 | 427 | 11886 |
| UBQLN2 | Ubqln2 | 29978 | 54609 |
| S100A4 | S100a4 | 6275 | 20198 |
| RTCA | Rtca | 8634 | 66368 |
| PSMD8 | Psmd8 | 5714 | 57296 |
| THY1 | Thy1 | 7070 | 21838 |
| PMM2 | Pmm2 | 5373 | 54128 |
| SRSF1 | Srsf1 | 6426 | 110809 |
| PACSIN2 | Pacsin2 | 11252 | 23970 |
| UBE2V2 | Ube2v2 | 7336 | 70620 |
| BAG3 | Bag3 | 9531 | 29810 |
| NUCB1 | Nucb1 | 4924 | 18220 |
| TSTA3 | Tsta3 | 7264 | 22122 |
| RAB1B | Rab1b | 81876 | 76308 |
| NAPA | Napa | 8775 | 108124 |
| CASK | Cask | 8573 | 12361 |
| ATG7 | Atg7 | 10533 | 74244 |
| UFSP2 | Ufsp2 | 55325 | 192169 |
| SNRNP200 | Snrnp200 | 23020 | 320632 |
| VAPA | Vapa | 9218 | 30960 |
| CD9 | Cd9 | 928 | 12527 |
| NDUFA5 | Ndufa5 | 4698 | 68202 |
| MAPRE1 | Mapre1 | 22919 | 13589 |
| COPE | Cope | 11316 | 59042 |
| SH3BGRL2 | Sh3bgrl2 | 83699 | 212531 |
| COL4A1 | Col4a1 | 1282 | 12826 |
| AHCY | Ahcy | 191 | 269378 |
| EPDR1 | Epdr1 | 54749 | 105298 |
| HIST1H1C | Hist1h1c | 3006 | 50708 |
| SLC4A1 | Slc4a1 | 6521 | 20533 |
| RPL12 | Rpl12 | 6136 | 269261 |
| NAMPT | Nampt | 10135 | 59027 |
| PAFAH1B2 | Pafah1b2 | 5049 | 18475 |
| HSPA9 | Hspa9 | 3313 | 15526 |
| PGRMC2 | Pgrmc2 | 10424 | 70804 |
| ACACB | Acacb | 32 | 100705 |
| BDH2 | Bdh2 | 56898 | 69772 |
| GMFG | Gmfg | 9535 | 63986 |
| GMFG | Gm9835 | 9535 | 100040018 |
| BZW1 | Bzw1 | 9689 | 66882 |
| CUTA | Cuta | 51596 | 67675 |
| HADH | Hadh | 3033 | 15107 |
| DDX17 | Ddx17 | 10521 | 67040 |
| MPI | Mpi | 4351 | 110119 |
| PITHD1 | Pithd1 | 57095 | 66193 |
| MTMR2 | Mtmr2 | 8898 | 77116 |
| GSTP1 | Gstp1 | 2950 | 14870 |
| GSTP1 | Gstp2 | 2950 | 14869 |
| GSTP1 | Gm3934 | 2950 | 100042625 |
| ACOT13 | Acot13 | 55856 | 66834 |
| APMAP | Apmap | 57136 | 71881 |
| ANXA5 | Anxa5 | 308 | 11747 |
| CSPG4 | Cspg4 | 1464 | 121021 |
| ACOX2 | Acox2 | 8309 | 93732 |
| PSMD7 | Psmd7 | 5713 | 17463 |
| SFPQ | Sfpq | 6421 | 71514 |
| DNAJB1 | Dnajb1 | 3337 | 81489 |
| KNG1 | Kng1 | 3827 | 16644 |
| KNG1 | Kng2 | 3827 | 385643 |
| RAB18 | Rab18 | 22931 | 19330 |
| DDX39B | Ddx39b | 7919 | 53817 |
| ACTR1B | Actr1b | 10120 | 226977 |
| DNAJA2 | Dnaja2 | 10294 | 56445 |
| ACOX3 | Acox3 | 8310 | 80911 |
| SP1 | Sp1 | 6667 | 20683 |
| C9orf64 | 2210016F16Rik | 84267 | 70153 |
| RSU1 | Rsu1 | 6251 | 20163 |
| EFHD2 | Efhd2 | 79180 | 27984 |
| HINT2 | Hint2 | 84681 | 68917 |
| PDHB | Pdhb | 5162 | 68263 |
| NIT2 | Nit2 | 56954 | 52633 |
| ATP5L | Atp5l | 10632 | 27425 |
| ANXA3 | Anxa3 | 306 | 11745 |
| CBR3 | Cbr3 | 874 | 109857 |
| HEXB | Hexb | 3074 | 15212 |
| FABP5 | Fabp5 | 2171 | 16592 |
| GPX1 | Gpx1 | 2876 | 14775 |
| LRRFIP1 | Lrrfip1 | 9208 | 16978 |
| SNRPE | LOC102632439 | 6635 | 102632439 |
| GSTK1 | Gstk1 | 373156 | 76263 |
| MRPL12 | Mrpl12 | 6182 | 56282 |
| AP1G1 | Ap1g1 | 164 | 11765 |
| GCHFR | Gchfr | 2644 | 320415 |
| EZR | Ezr | 7430 | 22350 |
| CMA1 | Cma1 | 1215 | 17228 |
| RANBP1 | Ranbp1 | 5902 | 19385 |
| AKR1A1 | Akr1a1 | 10327 | 58810 |
| NDUFA8 | Ndufa8 | 4702 | 68375 |
| ALDOC | Aldoc | 230 | 11676 |
| PCCA | Pcca | 5095 | 110821 |
| TFG | Tfg | 10342 | 21787 |
| EPHX2 | Ephx2 | 2053 | 13850 |
| SNRPD2 | Snrpd2 | 6633 | 107686 |
| CTPS2 | Ctps2 | 56474 | 55936 |
| IQGAP1 | Iqgap1 | 8826 | 29875 |
| SNRPB2 | Snrpb2 | 6629 | 20639 |
| XRN2 | Xrn2 | 22803 | 24128 |
| PSMA4 | Psma4 | 5685 | 26441 |
| PRKACA | Prkaca | 5566 | 18747 |
| EMC2 | Emc2 | 9694 | 66736 |
| GPI | Gpi1 | 2821 | 14751 |
| PSMA3 | Psma3 | 5684 | 19167 |
| HSP90B1 | Hsp90b1 | 7184 | 22027 |
| TES | Tes | 26136 | 21753 |
| RPN2 | Rpn2 | 6185 | 20014 |
| FNTB | Fntb | 2342 | 110606 |
| TUSC5 | Tusc5 | 286753 | 237858 |
| LSS | Lss | 4047 | 16987 |
| ILF3 | Ilf3 | 3609 | 16201 |
| MSRA | Msra | 4482 | 110265 |
| HNRNPR | Hnrnpr | 10236 | 74326 |
| SEPHS1 | Sephs1 | 22929 | 109079 |
| WDR61 | Wdr61 | 80349 | 66317 |
| ADHFE1 | Adhfe1 | 137872 | 76187 |
| ACADVL | Acadvl | 37 | 11370 |
| ACTR2 | Actr2 | 10097 | 66713 |
| BCL2L13 | Bcl2l13 | 23786 | 94044 |
| USP14 | Usp14 | 9097 | 59025 |
| QKI | Qk | 9444 | 19317 |
| CFH | Cfh | 3075 | 12628 |
| NDUFV2 | Ndufv2 | 4729 | 72900 |
| EIF3E | Eif3e | 3646 | 16341 |
| YAP1 | Yap1 | 10413 | 22601 |
| MATR3 | Matr3 | 9782 | 17184 |
| INF2 | Inf2 | 64423 | 70435 |
| HSPBP1 | Hspbp1 | 23640 | 66245 |
| SEC23IP | Sec23ip | 11196 | 207352 |
| DDAH2 | Ddah2 | 23564 | 51793 |
| L3HYPDH | L3hypdh | 112849 | 67217 |
| GLOD4 | Glod4 | 51031 | 67201 |
| CBX3 | Cbx3 | 11335 | 12417 |
| PPP2CA | Ppp2ca | 5515 | 19052 |
| AIFM1 | Aifm1 | 9131 | 26926 |
| GLUL | Glul | 2752 | 14645 |
| FABP3 | Fabp3 | 2170 | 14077 |
| HEBP1 | Hebp1 | 50865 | 15199 |
| ELAVL1 | Elavl1 | 1994 | 15568 |
| PSMB8 | Psmb8 | 5696 | 16913 |
| GNB1 | Gnb1 | 2782 | 14688 |
| GLS | Gls | 2744 | 14660 |
| CDH13 | Cdh13 | 1012 | 12554 |
| EPHX1 | Ephx1 | 2052 | 13849 |
| PABPC1 | Pabpc1 | 26986 | 18458 |
| COX17 | Cox17 | 10063 | 12856 |
| GDA | Gda | 9615 | 14544 |
| IVD | Ivd | 3712 | 56357 |
| APOD | Apod | 347 | 11815 |
| TRIM25 | Trim25 | 7706 | 217069 |
| ACBD3 | Acbd3 | 64746 | 170760 |
| HNRNPH3 | Hnrnph3 | 3189 | 432467 |
| ACAT2 | Acat3 | 39 | 224530 |
| ACAT2 | Acat2 | 39 | 110460 |
| TOM1L2 | Tom1l2 | 146691 | 216810 |
| F11R | F11r | 50848 | 16456 |
| DCTN2 | Dctn2 | 10540 | 69654 |
| CNN2 | Cnn2 | 1265 | 12798 |
| ARCN1 | Arcn1 | 372 | 213827 |
| RAP1A | Rap1a | 5906 | 109905 |
| MGLL | Mgll | 11343 | 23945 |
| RABGGTB | Rabggtb | 5876 | 19352 |
| AGO1 | Ago1 | 26523 | 236511 |
| IMPAD1 | Impad1 | 54928 | 242291 |
| GFM1 | Gfm1 | 85476 | 28030 |
| REEP5 | Reep5 | 7905 | 13476 |
| ANXA4 | Anxa4 | 307 | 11746 |
| TPSAB1 | Tpsb2 | 7177 | 17229 |
| RPIA | Rpia | 22934 | 19895 |
| TXNRD2 | Txnrd2 | 10587 | 26462 |
| ATP5F1 | Atp5f1 | 515 | 11950 |
| PSMB3 | Psmb3 | 5691 | 26446 |
| FBN1 | Fbn1 | 2200 | 14118 |
| HEBP2 | Hebp2 | 23593 | 56016 |
| RGN | Rgn | 9104 | 19733 |
| ALDH3A2 | Aldh3a2 | 224 | 11671 |
| CRAT | Crat | 1384 | 12908 |
| CD151 | Cd151 | 977 | 12476 |
| RTN1 | Rtn1 | 6252 | 104001 |
| COTL1 | Cotl1 | 23406 | 72042 |
| PTER | Pter | 9317 | 19212 |
| LARS | Lars | 51520 | 107045 |
| ERAP1 | Erap1 | 51752 | 80898 |
| MAPK3 | Mapk3 | 5595 | 26417 |
| STBD1 | Stbd1 | 8987 | 52331 |
| STX7 | Stx7 | 8417 | 53331 |
| RBKS | Rbks | 64080 | 71336 |
| HNRNPAB | Hnrnpab | 3182 | 15384 |
| HIBADH | Hibadh | 11112 | 58875 |
| SNCG | Sncg | 6623 | 20618 |
| SFN | Sfn | 2810 | 55948 |
| CAPNS1 | Capns1 | 826 | 12336 |
| UBE2K | Ube2k | 3093 | 53323 |
| MYL12A | Myl12b | 10627 | 67938 |
| MAPT | Mapt | 4137 | 17762 |
| COX6A1 | Cox6a1 | 1337 | 12861 |
| GARS | Gars | 2617 | 353172 |
| ISYNA1 | Isyna1 | 51477 | 71780 |
| BLVRB | Blvrb | 645 | 233016 |
| PCYOX1 | Pcyox1 | 51449 | 66881 |
| HP | Hp | 3240 | 15439 |
| GSTO1 | Gsto1 | 9446 | 14873 |
| SCO1 | Sco1 | 6341 | 52892 |
| CAPN1 | Capn1 | 823 | 12333 |
| HMBS | Hmbs | 3145 | 15288 |
| GPNMB | Gpnmb | 10457 | 93695 |
| SNTB2 | Sntb2 | 6645 | 20650 |
| PGRMC1 | Pgrmc1 | 10857 | 53328 |
| SH3KBP1 | Sh3kbp1 | 30011 | 58194 |
| IARS2 | Iars2 | 55699 | 381314 |
| QTRT1 | Qtrt1 | 81890 | 60507 |
| DYNC1LI2 | Dync1li2 | 1783 | 234663 |
| HDHD3 | Hdhd3 | 81932 | 72748 |
| C11orf68 | AI837181 | 83638 | 107242 |
| TLN2 | Tln2 | 83660 | 70549 |
| EPB41L1 | Epb4.1l1 | 2036 | 13821 |
| GCSH | Gcsh | 2653 | 68133 |
| USMG5 | Usmg5 | 84833 | 66477 |
| WDR13 | Wdr13 | 64743 | 73447 |
| GSTZ1 | Gstz1 | 2954 | 14874 |
| ECI2 | Eci2 | 10455 | 23986 |
| CLCC1 | Clcc1 | 23155 | 229725 |
| DLGAP4 | Dlgap4 | 22839 | 228836 |
| PPP1R7 | Ppp1r7 | 5510 | 66385 |
| RPSA | Rpsa | 3921 | 16785 |
| AP1B1 | Ap1b1 | 162 | 11764 |
| TCERG1 | Tcerg1 | 10915 | 56070 |
| NAGLU | Naglu | 4669 | 27419 |
| RPS12 | Rps12 | 6206 | 20042 |
| CAST | Cast | 831 | 12380 |
| PRKCSH | Prkcsh | 5589 | 19089 |
| SEC24C | Sec24c | 9632 | 218811 |
| GNAS | Gnas | 2778 | 14683 |
| CES2 | Ces2h | 8824 | 436059 |
| DCTD | Dctd | 1635 | 320685 |
| VARS | Vars | 7407 | 22321 |
| PLOD2 | Plod2 | 5352 | 26432 |
| PTGES3 | Gm9769 | 10728 | 100043508 |
| PTGES3 | Ptges3 | 10728 | 56351 |
| ACAA1 | Acaa1a | 30 | 113868 |
| ABHD5 | Abhd5 | 51099 | 67469 |
| PPP1CC | Gm5601 | 5501 | 434233 |
| CYB5R1 | Cyb5r1 | 51706 | 72017 |
| ALDH1A1 | Aldh1a1 | 216 | 11668 |
| METAP2 | Metap2 | 10988 | 56307 |
| GAPDH | Gapdh | 2597 | 14433 |
| GAPDH | Gm20899 | 2597 | 100042025 |
| PPP1R1B | Ppp1r1b | 84152 | 19049 |
| TGFBI | Tgfbi | 7045 | 21810 |
| ACADSB | Acadsb | 36 | 66885 |
| UPF1 | Upf1 | 5976 | 19704 |
| ADH5 | Adh5 | 128 | 11532 |
| DBI | Dbi | 1622 | 13167 |
| PPM1A | Ppm1a | 5494 | 19042 |
| COL6A2 | Col6a2 | 1292 | 12834 |
| MTCH2 | Mtch2 | 23788 | 56428 |
| OGN | Ogn | 4969 | 18295 |
| TUBB4B | Tubb4b | 10383 | 227613 |
| SYNM | Synm | 23336 | 233335 |
| HNRNPU | Hnrnpu | 3192 | 51810 |
| WDR77 | Wdr77 | 79084 | 70465 |
| XPNPEP1 | Xpnpep1 | 7511 | 170750 |
| MGST1 | Mgst1 | 4257 | 56615 |
| CRKL | Crkl | 1399 | 12929 |
| USP7 | Usp7 | 7874 | 252870 |
| ALDH7A1 | Aldh7a1 | 501 | 110695 |
| AKR1D1 | Akr1d1 | 6718 | 208665 |
| GDI2 | Gdi2 | 2665 | 14569 |
| CRYZ | Cryz | 1429 | 12972 |
| XRCC5 | Xrcc5 | 7520 | 22596 |
| PSMB7 | Psmb7 | 5695 | 19177 |
