# Supplementary material for: Cell-free fat extract improves ovarian function and fertility in mice with premature ovarian insufficiency
Source: Stem Cell Res Ther. 2022 Jul 16;13:320. doi: 10.1186/s13287-022-03012-w (PMC9288692; doi:10.1186/s13287-022-03012-w)
Supplement: Supplementary file 5 — Additional file 5: Table S3. Apoptosis related proteins in CEFFE. [file 13287_2022_3012_MOESM5_ESM.docx]

**Cell-free Fat Extract Improves Ovarian Function and Fertility in Mice with Premature Ovarian Insufficiency**

**Additional file 5**

**Supplementary Table S3. Apoptosis related proteins in CEFFE**

| Gene name |
| --- |
| MTCH2 |
| RPS3 |
| DFFA |
| AIFM1 |
| HNRNPK |
| TPT1 |
| CLU |
| CD44 |
| MIF |
| HTRA2 |
| NONO |
| PARP1 |
| SOD1 |
| NOL3 |
| UBQLN1 |
| HSPH1 |
| HSPB1 |
| PARK7 |
| SOD2 |
| SFPQ |
| GPX1 |
| P4HB |
| GSTP1 |
| NRP1 |
| PAK2 |
| ITGA6 |
| PEA15 |
| CAV1 |
| PPP2R1A |
| RELA |
| FGG |
| PDIA3 |
| LGALS3 |
| CTTN |
| RIPK1 |
| HSPA1B |
| GCLC |
| PML |
| ITGAV |
| YAP1 |
| PPP1CA |
| FGA |
| CTNNA1 |
| PYCARD |
| YWHAQ |
| YWHAG |
| YWHAH |
| SFN |
| YWHAB |
| DYNLL1 |
| DYNLL2 |
| PPP3R1 |
| YWHAE |
| YWHAZ |
| NMT1 |
| SERPINB9 |
| ARL6IP1 |
| POR |
| GPI |
| SNCA |
| PRDX5 |
| LAMTOR5 |
| AQP1 |
| PRDX3 |
| ACAA2 |
| CD248 |
| SORT1 |
| COL18A1 |
| EIF2S1 |
| AKR1C3 |
| VPS35 |
| FIS1 |
| NDUFA13 |
| RACK1 |
| TXNDC12 |
| PSMD10 |
| SLC9A3R1 |
| RRM2B |
| OPA1 |
| HYOU1 |
| CTSC |
| VDAC2 |
| BCAP31 |
| ERP29 |
| S100A9 |
| S100A8 |
| NDUFS3 |
| SLC25A5 |
| GNB1 |
| BLOC1S2 |
| BAG6 |
| UBE2K |
| ERO1A |
| ANXA1 |
| ADIPOQ |
| ILK |
| OXR1 |
| LRP1 |
| CDC42 |
| AARS |
| UBE2M |
| PPT1 |
| ITGA1 |
| UBE2V2 |
| SET |
| NES |
| NAE1 |
| HSPD1 |
| MECP2 |
| HSP90AB1 |
| CORO1A |
| G6PD |
| NQO2 |
| HSPB6 |
| ADA |
| TXNDC5 |
| PDIA6 |
| RAC1 |
| CD36 |
| TCP1 |
| BAG3 |
| NACA |
| ARL6IP5 |
| DIABLO |
| PDCD6 |
| VCP |
| MAPT |
| BCL2L13 |
| ANP32B |
| HIP1 |
| HSPE1 |
| PPM1F |
| CAST |
| NDUFS1 |
| HK2 |
| SLC25A4 |
| PPP2CB |
| GGCT |
| AIFM2 |
| CAMK2D |
