# Supplementary material for: Cell-free fat extract improves ovarian function and fertility in mice with premature ovarian insufficiency
Source: Stem Cell Res Ther. 2022 Jul 16;13:320. doi: 10.1186/s13287-022-03012-w (PMC9288692; doi:10.1186/s13287-022-03012-w)
Supplement: Supplementary file 6 — Additional file 6: Table S4. Proliferation related proteins in CEFFE. [file 13287_2022_3012_MOESM6_ESM.docx]

**Cell-free Fat Extract Improves Ovarian Function and Fertility in Mice with Premature Ovarian Insufficiency**

**Additional file 6**

**Supplementary Table S4. Proliferation related proteins in CEFFE**

| Gene name |
| --- |
| GSTP1 |
| FTH1 |
| S100A6 |
| FNTB |
| MIF |
| ANXA2 |
| PML |
| UTS2 |
| EGFR |
| FN1 |
| DDR2 |
| IFI30 |
| AQP1 |
| DDX39B |
| CNN1 |
| TPM1 |
| NQO2 |
| STAT6 |
| MAPK1 |
| ILK |
| CYBA |
| CDH13 |
| AIF1 |
| SF1 |
| ADIPOQ |
| XRCC6 |
| AKR1B1 |
| NAMPT |
| STAT1 |
| APOD |
| XRCC5 |
| NDRG2 |
| CTNNBIP1 |
| OGN |
| EEF2 |
| CLU |
| HIST1H4A |
| NASP |
| H3F3A |
| NPM1 |
| RUVBL1 |
| RBBP7 |
| LRP1 |
| SNCA |
| SLC9A3R1 |
| FBN1 |
| CFL1 |
| CRK |
| STAT5B |
| STAT3 |
| MAPK3 |
| PXN |
| PTK2 |
| RAB1A |
| LTBP4 |
| STAM |
| CDC42 |
| EPS15 |
| HGS |
| EPS15L1 |
| RAB7A |
| ITGA1 |
| GRB2 |
| SNX6 |
| UBA52 |
| SH3KBP1 |
| COL1A2 |
| FERMT2 |
| LIMS1 |
| NEDD8 |
| UBE2M |
| CAV1 |
| FKBP1A |
| COL4A2 |
| USP15 |
| F11R |
| FNTA |
| YES1 |
| PPM1A |
| PARP1 |
| ZYX |
| SNRNP70 |
| COL1A1 |
| FMOD |
| RHOA |
| NRP1 |
| MYO1C |
| HSPB1 |
| HRG |
| DCN |
| PDCD6 |
| PAK2 |
| RAC1 |
| CYFIP1 |
| ACTG1 |
| HSP90AA1 |
| ABI1 |
| NCKAP1 |
| ITGAV |
| ACTB |
| TPR |
| NCL |
| IQGAP1 |
| SORT1 |
| ARPC3 |
| CORO1A |
| RAP1A |
| HSPA5 |
| EHD1 |
| RELA |
| GCLC |
| CORO1B |
| RDX |
| HIP1 |
| CACYBP |
| PNPT1 |
| NME2 |
| ARF4 |
| EFEMP1 |
| PTPN11 |
| RAB21 |
| DPYSL2 |
| IST1 |
| MAP1B |
| MAPT |
| GOLGA4 |
| RTN4 |
| TWF2 |
| PAFAH1B1 |
| CTTN |
| G6PD |
